# The pyruvate dehydrogenase complex in concert with the DNA/RNA-binding protein YBX1 regulates cell senescence and tumorigenesis

**DOI:** 10.1016/j.jbc.2025.110585

**Published:** 2025-08-12

**Authors:** Huan Chen, Jing Shi, Qiuping Wang, Kunming Liang, Shaojun Pei, Yi Zhang, Xiang Shi, Jing Lv, Tian Xia, Di Chen, Yegang Ma, Hong-Xu Liu, Hai-long Piao

**Affiliations:** 1 State Key Laboratory of Phytochemistry and Natural Medicines, Dalian Institute of Chemical Physics, Chinese Academy of Sciences, Dalian, China; 2 Department of Biochemistry & Molecular Biology, School of Life Sciences, China Medical University, Shenyang, China; 3 University of Chinese Academy of Sciences, Beijing, China; 4 Department of Thoracic Surgery, Cancer Hospital of Dalian University of Technology, Liaoning Cancer Hospital & Institute, Shenyang, China

**Keywords:** pyruvate, PDHA1, YBX1, senescence, cancer

## Abstract

The activation of oncogenes is often accompanied by metabolic adaptations. The DNA/RNA-binding protein, Y-box binding protein 1 (YBX1), a well-known oncogene, is hyperactivated in nearly all cancer types. However, the metabolic hallmarks associated with YBX1 and the underlying mechanisms remain poorly understood. Here, we demonstrate that YBX1 sustains the activity of the pyruvate dehydrogenase (PDH) complex by regulating its catalytic alpha subunit, PDHA1. Mechanistically, YBX1 downregulates the expression of PDH kinases 1 and 4, leading to enhanced PDHA1 activity. Consequently, YBX1 deficiency results in significant impairment of pyruvate oxidation. Moreover, we found that YBX1, in conjunction with PDH activation, promotes epithelial cell senescence and suppresses cancer cell proliferation. These findings indicate that PDH activation imposes a constraint on the tumorigenic potential of YBX1.

Flexibility is a pivotal feature of metabolism, and all organisms rely on this flexibility to adapt to the ever-changing environment for survival. Compared with normal cells, the metabolic flexibility of cancer cells is often reduced (1, 2), as tumor cells must acquire numerous irreversible adaptive metabolic changes, that is, metabolic remodeling, to maintain survival or proliferation during the onset and development of cancer (3), such as increased glycolysis (4), glutamine dependence (5), and elevated arginine levels (6). Among the adaptive metabolic changes of cancer cells, intermediates of tricarboxylic acid (TCA) cycle and related metabolites were frequently dysregulated because of mutations, deletions, or amplifications of tumor suppressor genes and proto-oncogenes (2, 7). PDHA1, a component of pyruvate dehydrogenase (PDH) complex (PDHc), is a rate-limiting enzyme of the complex. PDHc catalyzes the pyruvate into acetyl CoA, thereby, entry into TCA cycle by transforming as citrate in mitochondrial matrix (8). On one hand, decreased PDHA1 activity promotes NAD^+^ regeneration and confers cell resistance to oncogene-induced senescence (9, 10). On the other hand, PDHA1 is required for the initiation of leukemia and prostate cancer (11, 12). Thus, the function of PDH in cancer development appears to be context dependent.

Y-box binding protein 1 (YBX1) is a multifunctional DNA/RNA-binding protein, distinguished by its conserved cold shock domain (13). YBX1 is highly expressed during early embryonic development; its absence leads to lethality in late embryonic development (14). In aged mice, YBX1 is notably absent from most tissues, with the notable exception of liver tissue (15). Conversely, YBX1 is overexpressed in most cancer cells, where it transcriptionally or translationally activates a number of oncogenes to promote proliferation, drug resistance, and metastasis (16). YBX1 is indirectly involved in cell metabolism by regulating hypoxia-inducible factor 1-alpha (HIF1α) and MYC expression (17, 18, 19). In addition, our group recently reported that YBX1 localizes to the mitochondrial inner membrane space in specific cancer cells, where it binds to mitochondrial pyruvate carrier (MPC), thereby inhibiting pyruvate uptake to promote cell metastasis (20). However, the characteristics of the metabolic adaptation induced by YBX1 are not well understood in detail.

In the present study, using an unbiased mass spectrometry–based phosphoproteomics assay, we systematically elucidated the interplay between YBX1 and the perturbations in protein phosphorylation that govern metabolic regulation. We observed that several enzyme phosphorylation sites were markedly altered upon YBX1 deficiency, and these enzymes are involved in crucial metabolic pathways, such as nucleic acid synthesis, fatty acid metabolism, and TCA cycle. Among them, the phosphorylation of PDHA1 at s232, s293, and s300 sites was significantly increased nearly 30-fold in YBX1 knockdown cells, suggesting that YBX1 is important for maintaining PDHA1 activity. Here, we uncover a previously undescribed mechanism by which YBX1 regulates PDHA1 activity and the role of the YBX1–PDHA1 axis in cancer cells.

## Results

### PDHA1 activation required YBX1

**Figure 1:**
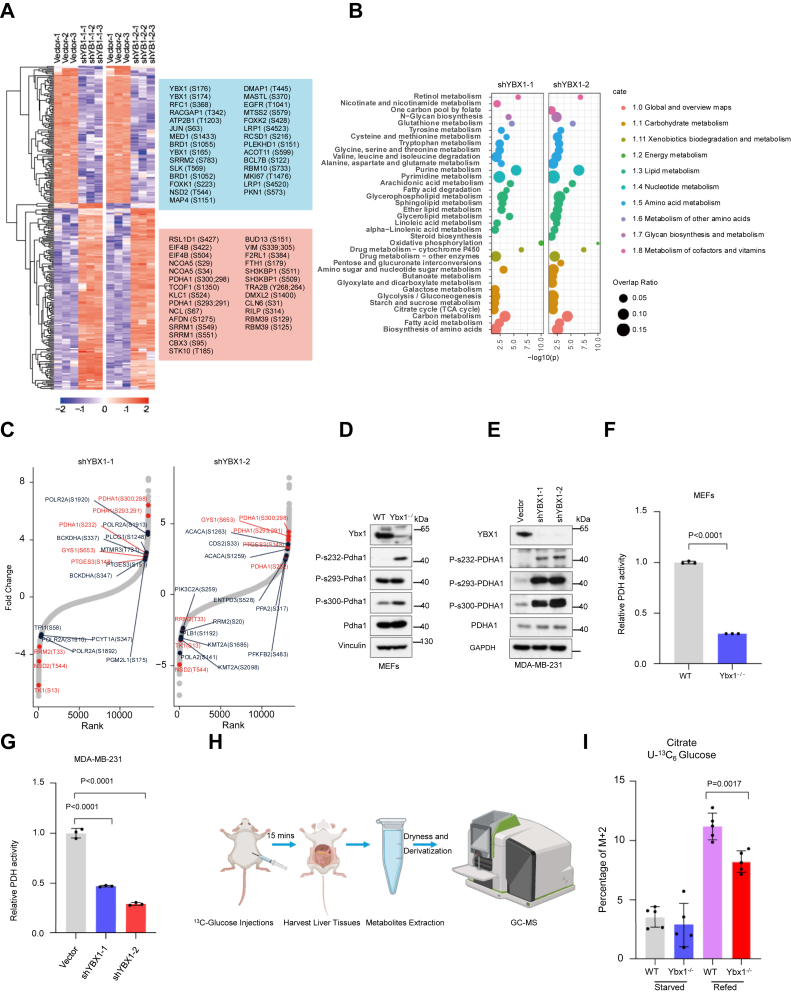
**YBX1 is important for PDHA1 activation**. *A*, heat map analysis of regulated phosphosites (*q* < 0.05, difference >2) in control and shRNA-mediated YBX1 knockdown MDA-MB-231 cells. Panel highlight shows the intersection of the top 100 coaltered phosphoproteins along with amino acid sites in YBX1 knockdown cells. Phosphosites in *pink box* mean upregulated, whereas the *blue box* means downregulated (shYBX1 *versus* vector). *B*, enriched metabolic signaling pathway analysis based on significantly altered phosphoproteins in YBX1 knockdown cells. *C*, ranking of phosphorylated phosphosites in two independent YBX1 shRNA-transduced MDA-MB-231 cells. The significantly coaltered phosphosites involved in metabolism were highlighted in *red*. *D*, WT and *Ybx1*^−/−^ MEFs were subjected to immunoblotting with indicated antibodies. *E*, immunoblotting analysis with indicated antibodies in MDA-MB-231 cells transduced with two independent shRNAs of YBX1. *F*, the lysates of WT and *Ybx1*^−/−^ MEFs were subjected to PDH enzymatic activity analysis (*n* = 3 biological independent samples). *G*, MDA-MB-231 cells transduced with two independent YBX1 shRNAs, and cell lysates were subjected to PDH enzymatic activity analysis (*n* = 3 biological independent samples). *H*, schematic diagram of [U-^13^C] glucose tracing in mice. *I*, after fasted-refed condition, mice were intraperitoneally injected with [U-^13^C] glucose (2 g/kg), and the ^13^C distribution of citrate was analyzed using GC–MS measurement from mice liver tissues. (*n* = 5 biological independent samples). Mice were pretrained with 12 h fasting and 12 h refeeding daily for 3 days before the experiment. On the last day, the refed mice were free to standard diet for 8 h and the other mice continued to fast for 8 h followed by further treatment. Starved: fasted for 20 h; Refed: fasted 12 h and refed for another 8 h. Data in (*F*), (*G*), and (*I*) are the mean of biological replicates from a representative experiment, and error bars indicate SD. Statistical significance was determined by a two-tailed, unpaired Student’s *t* test. The experiments in (*D*–*G*) were performed independently at least three times. MEF, mouse embryonic fibroblast; PDH, pyruvate dehydrogenase; YBX1, Y-box binding protein 1.

Protein phosphorylation widely regulates metabolic reprogramming in cells (21). To identify the regulatory relationship between YBX1 and cellular metabolism, we performed mass spectrometry–based phosphoproteomics assays in two independent YBX1 shRNA-transduced MDA-MB-231 and Cre-loxP-induced *Ybx1* knockout mouse embryonic fibroblasts (*Ybx1*^−/−^ MEFs) cells. We quantified 12,185 distinct phosphorylation sites on 3547 different proteins (Fig. 1*A*; Fig. S1, *A* and *B*). Of note, the processes of RNA splicing and Janus kinase-–signal transducer and activator of transcription signaling proteins were involved (Fig. 1*A*; Fig. S1, *A* and *B*), which was consistent with a previous report (22). The pathway enrichment analysis showed that metabolic processes were altered upon YBX1 inactivation, such as oxidation phosphorylation, TCA cycle, and lipid metabolism (Fig. 1*B*). Among them, the top 20 ranked phosphosites were related to metabolic pathways, and we observed that eight phosphosites from six proteins were commonly altered in two distinct YBX1 shRNA knockdown MDA-MB-231 cells (Fig. 1*C*). Indeed, RRM2 and TK1 were decreased by YBX1 inactivation, which is involved in nucleotide metabolism in liver cancer cells (23). Importantly, the three phosphosites (s232, s293, and s300) of PDHA1 were highly elevated in YBX1-downregulated MDA-MB-231 cells and *Ybx1*^−/−^ MEFs (Fig. 1*C*; Fig. S1*C*).

The elevated PDHA1 phosphorylation inhibits PDHc enzymatic activity and subsequently inhibits pyruvate mitochondrial oxidation (8). Indeed, YBX1 inactivation increased PDHA1 phosphorylation and suppressed PDH activity (Fig. 1, *D*–*G*; Fig. S1, *D* and *E*). Next, we generated YBX1 liver-specific knockout mice by crossing Ybx1^*flox/flox*^ with Alb-Cre strain mice. In the mouse liver, fasting followed by refeeding decreased PDHA1 phosphorylation (Fig. S1*F*). We found that PDHA1 phosphorylation increased in *Ybx1*^−/−^ mouse livers compared with WT livers under fasted-refed conditions (Fig. S1*F*). In addition, the levels of pyruvate and lactate were increased under fasted-refed conditions; however, citrate levels were not affected under both fasting and refeeding conditions in *Ybx1*^−/−^ mouse livers (Fig. S1*G*), which may account for the metabolic flexibility in normal tissues (1). Consistently, [U-^13^C] glucose–traced citrate levels were reduced in *Ybx1*^−/−^ mouse livers under fasted-refed conditions but not in fasted conditions (Fig. 1, *H* and *I*). Taken together, these results indicate that YBX1 is critical for maintaining PDHc activity by regulating the phosphorylation of PDHA1 in mammalian cells.

### YBX1 deficiency inactivates PDHA1 and impairs pyruvate oxidation

**Figure 2:**
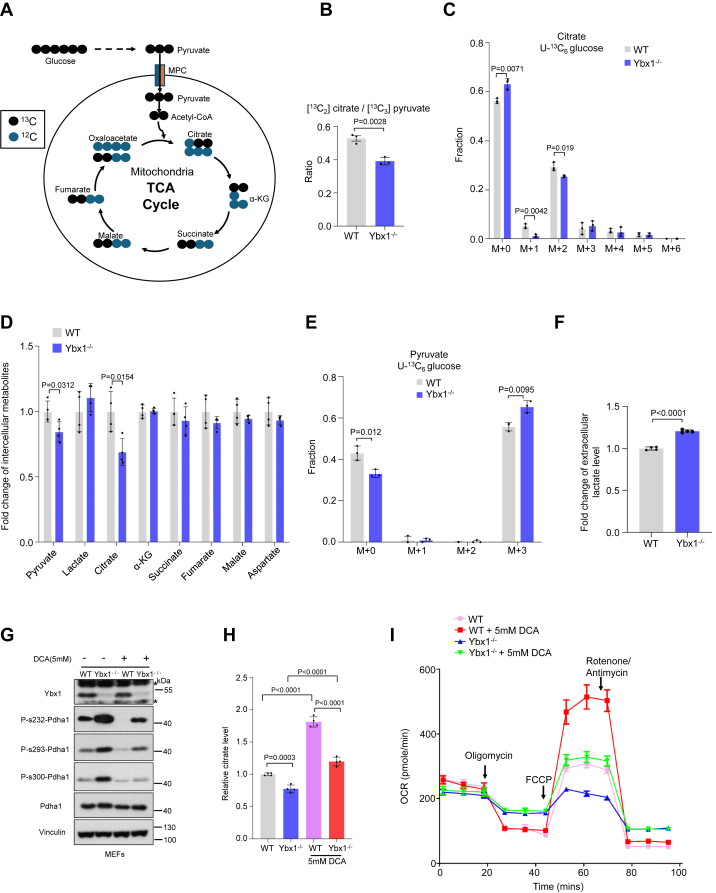
**YBX1 deficiency inactivates PDHA1 and impairs pyruvate oxidation**. *A*, diagram of ^13^C isotope-traced patterns from [U-^13^C] glucose. *B*, mass isotopolog analysis of [^13^C_2_] citrate/[^13^C_3_] pyruvate in WT and *Ybx*1^−/−^ MEFs incubated with [U-^13^C] glucose (*n* = 3 biological independent samples). *C*, mass isotopolog analysis of citrate in WT and *Ybx*1^−/−^ MEFs incubated with [U-^13^C] glucose (*n* = 3 biological independent samples). *D*, WT and *Ybx1*^−/−^ MEF cells were subjected to analysis of TCA cycle intermediate levels (*n* = 4 biological independent samples). *E*, mass isotopolog analysis of pyruvate in WT and *Ybx1*^−/−^ MEFs incubated with [U-^13^C] glucose (*n* = 3 biological independent samples). *F*, analysis of lactate levels in culture medium from WT and *Ybx*1^−/−^ MEFs (*n* = 4 biological independent samples). *G*–*I*, WT and *Ybx1*^−/−^ MEFs were treated with or without 5 mM DCA for 24 h, and immunoblotting analysis of indicated proteins (*G*), measurement of citrate levels (*n* = 4 biological independent samples) (*H*) and OCR (*n* = 4 biological independent samples) (*I*). “∗” means the nonspecific bands. Data in (*B*–*F*), (*H*), and (*I*) are the mean of biological replicates from a representative experiment, and error bars indicate SD. Statistical significance was determined by a two-tailed, unpaired Student’s *t* test. All experiments were repeated independently at least three times. DCA, dichloroacetate; MEF, mouse embryonic fibroblast; TCA, tricarboxylic acid; YBX1, Y-box binding protein 1.

To verify the function of YBX1 in mitochondrial pyruvate oxidation, we traced the metabolites of the TCA cycle by using [U-^13^C] glucose (Fig. 2*A*). YBX1 knockdown increased [U-^13^C] glucose–traced citrate and the ratio of [M+2] citrate to [M+3] pyruvate (Fig. S2, *A* and *B*), suggesting an increase in the activity of MPCs (20). However, the level of [M+2] citrate was reduced in YBX1 knockdown MDA-MB-231 cells (Fig. S2*A*). In addition, in MEFs, Ybx1 deletion decreased pyruvate catabolism (Fig. 2*B*), subsequently reduced [U-^13^C] glucose–traced citrate and intercellular citrate levels (Fig. 2, *C* and *D*). Ybx1 knockout also increased both the levels of pyruvate (M+3) and lactate (M+3) (Fig. 2*E*, Fig. S2*C*), whereas the ratio of lactate (M+3) to pyruvate (M+3) remained the same (Fig. S2*D*), indicating the lactate dehydrogenase activity was unaffected in *Ybx1*^−/−^ MEFs. In line with this, extracellular lactate levels increased in *Ybx1*^−/−^ MEFs (Fig. 2*F*), despite intercellular lactate remaining unchanged (Fig. 2*D*). It was worth noting that the pyruvate dehydrogenase kinase (PDK) inhibitor, dichloroacetate (DCA), partially decreased PDH phosphorylation and conversely restored citrate levels and the maximal oxygen consumption rate (OCR) in *Ybx1*^−/−^ MEFs (Fig. 2, *G*–*I*). Finally, [U-13C] glucose–derived citrate was reduced in YBX1 knockdown HBE and MCF-10A cells (Fig. S2, *E* and *F*), indicating that the role of YBX1 in maintaining PDHA1 activity is a ubiquitous phenomenon. Collectively, our data demonstrate that YBX1 promotes mitochondrial pyruvate oxidation in MEF and human epithelial cells.

### YBX1 regulates PDK

**Figure 3:**
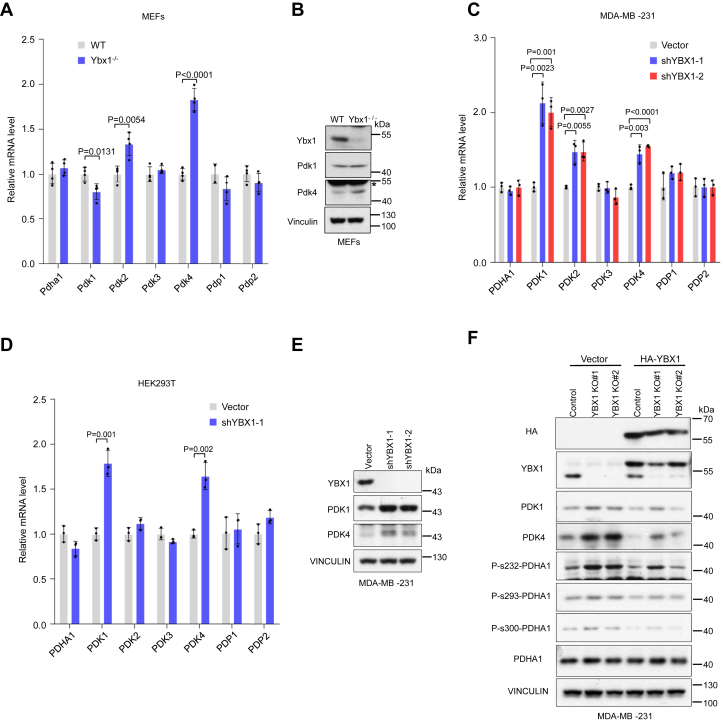
**Pyruvate dehydrogenase kinase mediates the effect of YBX1**. *A*, quantitative real-time PCR (qRT–PCR) analysis of indicated genes involved in PDH phosphorylation in WT and *Ybx1*^−/−^ MEF cells (*n* = 3 biological independent samples). *B*, immunoblotting analysis with indicated antibodies in WT and *Ybx1*^−/−^ MEF cells. *C*, qRT–PCR analysis of indicated genes involved in PDH phosphorylation in vector and shRNA-mediated knockdown MDA-MB-231 cells (*n* = 3 biological independent samples). *D*, qRT–PCR analysis of indicated genes involved in PDH phosphorylation in vector and shRNA-mediated knockdown HEK293T cells (*n* = 3 biological independent samples). *E*, immunoblotting analysis with indicated antibodies in vector and shRNA-mediated knockdown of MDA-MB-231 cells. *F*, immunoblotting analysis of indicated proteins in YBX1 knockout MDA-MB-231 cells and HA-tagged YBX1-restored cells. Data in (*A*), (*B*), and (*D*) are the mean of biological replicates from a representative experiment, and error bars indicate SD. Statistical significance was determined by a two-tailed, unpaired Student’s *t* test. The experiments in (*D*) were repeated twice, and all experiments were repeated independently at least three times. HEK293T, human embryonic kidney 293T cell line; MEF, mouse embryonic fibroblast; PDH, pyruvate dehydrogenase; YBX1, Y-box binding protein 1.

To further determine how YBX1 regulates PDHA1 activity, we examined the transcript levels of PDK1-4 and PDH phosphatase 1/2, which are key regulators of PDHA1 phosphorylation (8).We observed that phosphorylation of PDHA1 (P-s232, P-s293, and P-s300) and both transcripts and protein expression of Pdk4 were increased in *Ybx1*^−/−^ MEFs (Fig. 3, *A* and *B*). Moreover, YBX1 suppresses *Pdk4* expression accompanied by a reduction in its mRNA half-life but increases its cell growth in mouse 4T1 cells (Fig. S3, *A* and *B*). Notably, both the protein and mRNA levels of PDK1 and PDK4 were significantly upregulated in YBX1 knockdown MDA-MB-231 and human embryonic kidney 293T (HEK293T) cells, whereas other upstream factors of PDHA1 remained unaffected (Fig. 3, *B*–*E*). In addition, while YBX1 overexpression suppresses PDK4 transcripts accompanied by a reduction in its mRNA half-life and enhanced proliferation in HCT116 cells (Fig. S3, *C*–*E*). Since it was reported that shRNA-mediated YBX1 knockdown may result in unexpected off-target effect (19), we employed CRISPR-mediated genetic inactivation of *YBX1* to avoid the potential biases (Fig. S3*F*). YBX1 knockout increased both PDK1 and PDK4 protein expression; however, this increase was restored by overexpression of HA-tagged YBX1 using lentivirus transfection (Fig. 3*F*), suggesting that the inhibitory effect of YBX1 on PDK1 expression is authentic. We also observed that AKT–FOXO pathway, a known upstream of PDK4 (24, 25, 26), was not involved in YBX1–PDK4 regulation (Fig. S3*G*). We previously demonstrated that PDHA1 is activated by mammalian target of rapamycin complex 1 (mTORC1) with an unknown mechanism (27). In addition, both mTORC1 and mTORC2 are increased by YBX1 *via* promoting mLST8 protein expression (28). However, rapamycin treatment did not affect PDK1 and PDK4 protein expression in both control and YBX1 knockdown cells (Fig. S3*H*), indicating that the inhibitory effect of YBX1 on PDK1 and PDK4 expression is independent of mTORC1 signaling. Finally, PDK inhibitor, DCA, mitigated the impact of YBX1 on PDHA1 phosphorylation (Fig. S3*I*). These data demonstrated that YBX1 regulates PDHA1 activity in a manner dependent on PDK.

### YBX1 inhibits PDK1 expression

**Figure 4:**
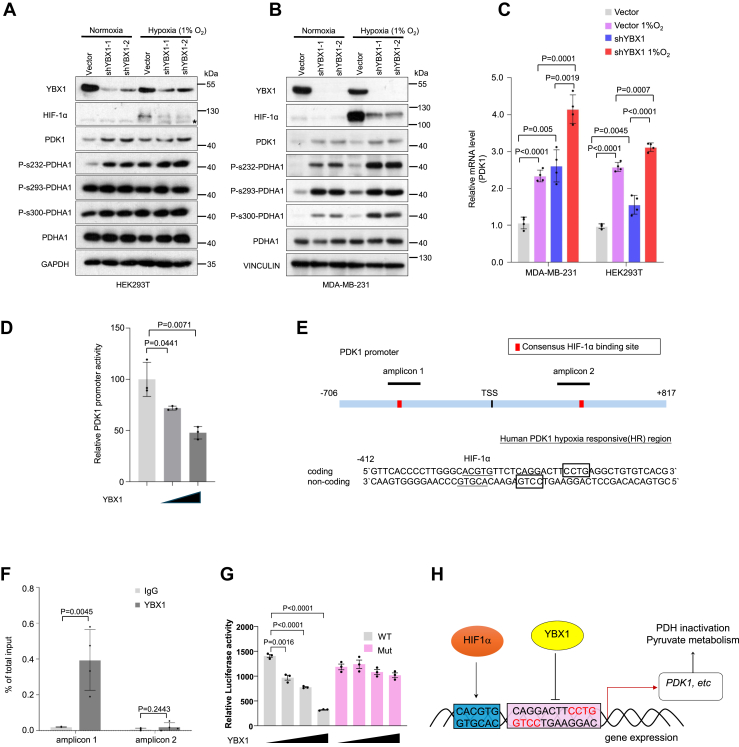
**YBX1 inhibits PDK1 expression**. *A* and *B*, immunoblotting analysis of indicated proteins in HEK293T (*A*) and MDA-MB-231 (*B*) cells transduced with two independent YBX1 shRNAs in normoxia or hypoxia condition (hypoxia treatment for 6 h). “∗” means the nonspecific bands. *C*, quantitative RT–PCR analysis of *PDK1* expression levels in HEK293T and MDA-MB-231 cells under normoxia or hypoxia condition (6 h) in indicated cells (*n* = 4 biological independent samples). *D*, luciferase activity assay of *PDK1* promoter activity in HEK293T cells transfected with different concentrations of YBX1 (*n* = 3 biological independent samples). *E*, schematic diagram of HRR in human *PDK1* promoter (*upper*) and the consensus sequence of 5′-CCTG-3′ around HRR (*below*). *F*, ChIP–quantitative PCR of YBX1 binding to the *PDK1* promoter HRR in MDA-MB-231 cells (*n* = 3 biological independent samples). *G*, luciferase activity in HEK293T cells cotransfected with WT or Mut PDK1 promoter reporters and increasing concentrations of YBX1 expression plasmid (0, 200, 500, and 1000 ng). Luciferase values represent mean ± SD of three independent experiments (∗*p* < 0.05, ∗∗*p* < 0.01, and ∗∗∗*p* < 0.001; two-way ANOVA with *post hoc* test). *H*, diagram of YBX1 binding HRR. Results in (*C*), (*D*), and (*F*) are the mean of biological replicates from a representative experiment, and error bars indicate SD. Statistical significance was determined by a two-tailed, unpaired Student’s *t* test. All other experiments were repeated independently at least three times. ChIP, chromatin immunoprecipitation; HEK293T, human embryonic kidney 293T cell line; HRR, hypoxia responsive region; PDH, pyruvate dehydrogenase; PDK1, pyruvate dehydrogenase kinase 1; YBX1, Y-box binding protein 1.

It was previously reported that HIF1α activates PDK1 transcription by directly binding to the *PDK1* promoter (29). In addition, YBX1 translationally activates the expression of HIF1α (17). These studies indicate that YBX1 expression may positively correlate with PDK1 expression. However, we observed contradictory results between YBX1 and PDK1 levels (Fig. 3, *C*–*E*). To determine the mechanism by which YBX1 inhibits PDK1, we investigated PDK1 and PDHA1 phosphorylation status under hypoxia conditions. HIF1α expression was reduced in YBX1-downregulated cells, both with and without hypoxia treatment (Fig. 4, *A* and *B*). However, both PDK1 protein and mRNA levels were increased in YBX1 knockdown cells, and hypoxia treatment further enhanced this increase, despite the reduced HIF1α expression (Fig. 4, *A*–*C*). Moreover, we confirmed the hypoxia treatment condition by detecting GLUT1 (30), a well-known HIF1α downstream gene in YBX1 knockdown HEK293T cells (Fig. S4*A*).

Since YBX1 is a DNA/RNA-binding protein that regulates mRNA degradation and transcription (13, 31), we next investigated whether YBX1 regulates either PDK1 mRNA stability or transcriptional activity. We observed that YBX1 inhibited the promoter activity of *PDK1* in a dose-dependent manner (Fig. 4*D*); nevertheless, the PDK1 mRNA levels remained stable and showed no obvious degradation within the 6 h in both YBX1 knockdown and control cells (Fig. S4*B*). Previously, two studies reported that YBX1 acts as a transcriptional repressor by binding to the consensus sequence of 5`-CCTG-3′ in the promoter of the human vascular endothelial growth factor hypoxia responsive (HR) region (HRR) (32, 33). We also identified two consensus elements within the HRR of the human *PDK1* promoter (Fig. 4*E*). Indeed, we found that YBX1 binds to amplicon 1 in the HRR of the human *PDK1* promoter but not bind to the CCTG deleted mutant HRR (Fig. 4, *F*–*H*). However, we did not detect a similar consensus sequence in the corresponding HRR of the mouse *Pdk1* promoter, which may explain why Pdk1 mRNA and protein levels remain unchanged in *Ybx1*^−/−^ MEFs and *Ybx1* knockdown 4T1 cells (Fig. 3, *A* and *B*; Fig. S4, *C*–*F*). Moreover, YBX1 and PDK1 showed a significant negative correlation in the human breast cancer protein expression database from the National Cancer Institute Clinical Proteomic Tumor Analysis Consortium, whereas there was no correlation between *Ybx1* and *Pdk1* in mouse mammary tumor tissues (34) (Fig. S4*G*). These data suggest that the transcriptional inhibition of PDK1 by YBX1 may be limited in human cells but not in mouse cells (Fig. 4*H*).

### YBX1 induces senescence by regulating PDHA1 activity

**Figure 5:**
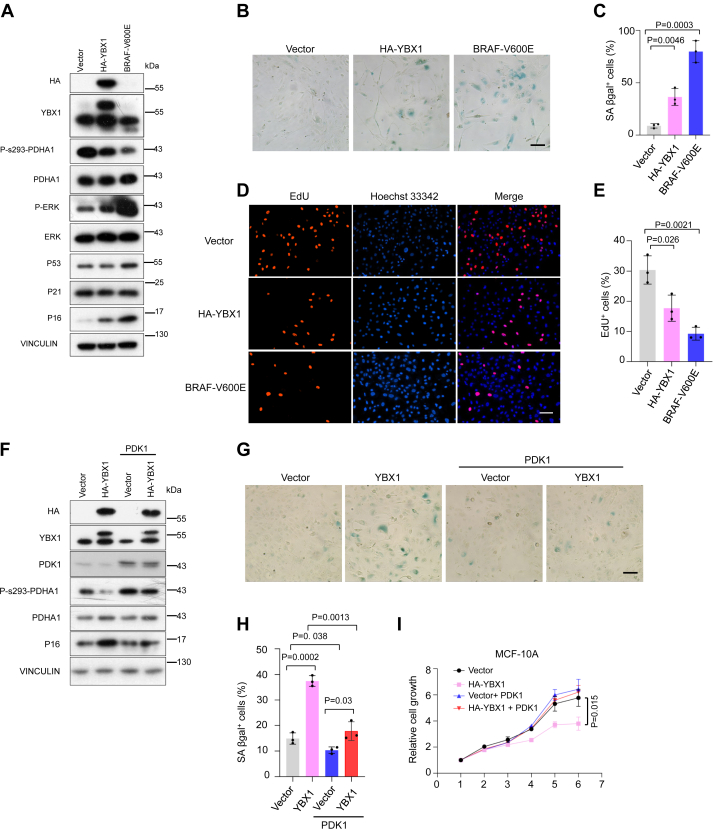
**YBX1 induces senescence by regulating PDHA1 activity in MCF-10A cells**. *A*, immunoblotting analysis of indicated proteins in MCF-10A cells transduced with HA-tagged YBX1 and BRAF-V600E for 11 days, *B* and *C*, representative images and quantification of SA-β-gal–positive cells. Scale bar represents 100 μm. *D* and *E*, EdU staining and quantification for analysis of the influences of YBX1 and BRAF-V600E on the proliferation of MCF-10A cells. Scale bar represents 100 μm. *F*, immunoblotting analysis of indicated proteins in MCF-10A cells transduced with HA-tagged YBX1 and PDK1 for 11 days. *G* and *H*, representative images and quantification of SA-β-gal–positive cells. Scale bar represents 100 μm. *I*, relative growth curve of vector, HA-tagged YBX1, PDK1, and HA-tagged YBX1 combined with PDK1 in MCF-10A cells (*n* = 3 biological independent samples). Results are the mean of biological replicates from a representative experiment, and error bars indicate SD. Statistical significance was determined by a two-tailed, unpaired Student’s *t* test. All experiments were repeated independently at least three times. EdU, 5-ethynyl-2′-deoxyuridine; PDK, pyruvate dehydrogenase kinase; SA-β-gal, senescence-associated β-galactosidase; YBX1, Y-box binding protein 1.

Although reducing YBX1 expression severely decreases cell proliferation among different cancer types (16), while hyperactivation of YBX1 also inhibited cell proliferation in MCF-10A (35, 36) as well as in liver cancer cells (37). Indeed, in MCF-10A cells, overexpression of YBX1 suppressed cell proliferation but enhanced the anchorage-independent growth in soft agar (Fig. S5, *A*–*D*). Notably, the overexpression of YBX1 significantly increased cell size in MCF-10A cells (Fig. S5, *E* and *F*), which is a well-known feature of senescence. Therefore, we using the oncogene BRAF-V600E as a positive control (9) and found that the activation of YBX1 and BRAF-V600E significantly decreased the phosphorylation of PDHA1 (Fig. 5*A*). Concurrently, it increased the senescence-associated β-galactosidase (SA-β-gal) activity and the levels of senescence marker of P16^INK4A^ expression (9) (Fig. 5, *A*–*C*), thereby contributing to cell growth arrest (Fig. 5, *D* and *E*). In addition, we noted that overexpression of YBX1 did not alter the expression levels of P53 and its downstream target, P21 (Fig. 5*A*).

PDH activation, leading to mitochondrial oxidative stress, plays a pivotal role in the oncogene-induced senescence (9). Consistent with this, the SA-β-gal activity and the levels of P16^INK4A^ protein significantly increased in PDK1 knockdown cells (Fig. S5, *G*–*I*). Furthermore, treatment with the PDK inhibitor, DCA, induced the activation of SA-β-gal activity (Fig. S5, *J* and *K*). These results suggest that YBX1-induced senescence may be dependent on PDHA1 activation.

Next, to determine the role of PDHA1 in YBX1-mediated senescence, we reconstituted the PDK1 expression in YBX1-overexpressed cells. PDK1 overexpression reduced the levels of P16^INK4A^ protein and the positivity of SA-β-gal in YBX1-overexpressing cells (Fig. 5, *F*–*H*). Moreover, the intercellular reactive oxygen species (ROS) level was elevated by YBX1 overexpression, but this elevation of ROS was diminished upon PDK1 restoration (Fig. S5*L*). Moreover, overexpression of PDK1 alleviated the proliferation arrest induced by YBX1 activation (Fig. 5*I*). Hence, these results indicate that YBX1 enforces of PDHA1 hyperactivation, thereby driving cellular senescence.

### PDHA1 activation constrains tumorigenic capacity of YBX1

**Figure 6:**
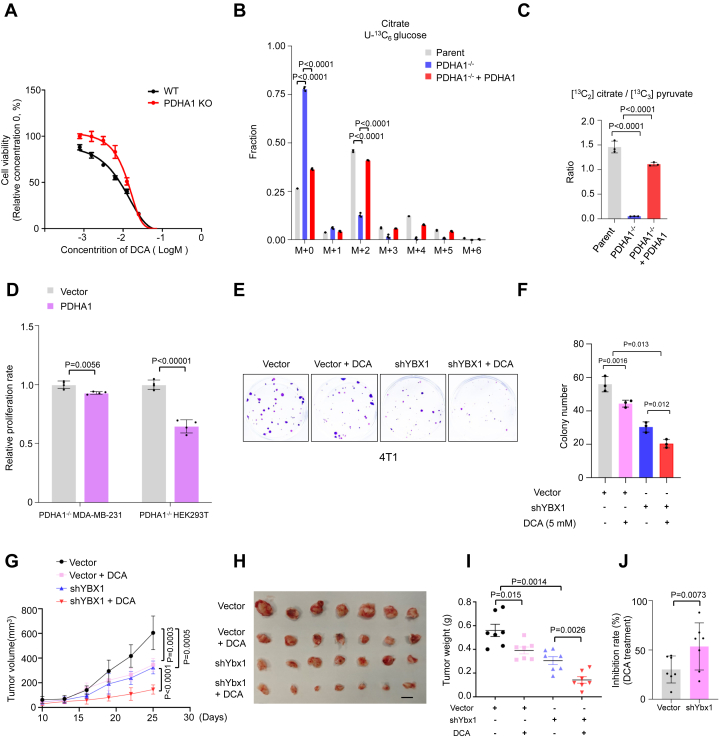
**PDHA1 activation constrain tumorigenic capacity of YBX1**. *A*, the cell viability of WT and PDHA1 knockout HEK293T cells treated with different concentrations (0, 0.78125, 1.5625, 3.125, 6.25, 12.5, 25, 50, and 100; mM) of DCA for 96 h (*n* = 3 biological independent samples). *B*, mass isotopolog analysis of citrate in parent, PDHA1^−/−^, and PDHA1 restoration PDHA1^−/−^ HEK293T cells incubated with [U-^13^C] glucose (*n* = 3 biological independent samples). *C*, mass isotopolog analysis of [^13^C_2_] citrate/[^13^C_3_] pyruvate in parent, PDHA1^−/−^, and PDHA1 restoration in PDHA1^−/−^ HEK293T cells incubated with [U-^13^C] glucose (*n* = 3 biological independent samples). *D*, the cell viability of PDHA1^−/−^ and PDHA1 restoration HEK293T and MDA-MB-231 cells (*n* = 3 biological independent samples). *E*, the cell colony formation analysis in vector and shRNA-mediated Ybx1 knockdown 4T1 cells combined with or without DCA (5 mM) treatment for 9 days (*n* = 3 biological independent samples). *F*, the cell colony number in (*E*). *G*, tumor volume was measured using caliper every 2 days, and tumor volume was calculated using standard formula *0*.*5 × L × W* (2), where L is the longest diameter and W is the shortest diameter. *H*, tumors in (*G*) were removed and pictured after treated with indicated drugs for 15 days, n = 7. *I*, tumor weight in mice from (*G*), n = 7. *J*, inhibition rate was calculated using tumor weights in (*I*), n = 7; inhibition rate = 1-DCA/vehicle. Results are the mean of biological replicates from a representative experiment, and error bars indicate SEM. Statistical significance was determined by a two-tailed, unpaired Student’s *t* test. All other experiments were repeated independently at least three times. DCA, dichloroacetate; HEK293T, human embryonic kidney 293T cell line; YBX1, Y-box binding protein 1.

To explore the role of YBX1-dependent PDHA1 activation in cancer cells, we first examined the effect of pyruvate catabolism on cell growth using PDK inhibitors. DCA, the most extensively investigated PDK inhibitor, elicits a dose-dependent inhibitory impact on cell growth within the millimole concentration range (Fig. 6*A*; Fig. S6*A*). In addition, cells lacking PDHA1 exhibited resistance to DCA treatment (Fig. 6*A*; Fig. S6*A*). The reconstitution of PDHA1 restored the M+2 citrate level derived from [U-^13^C] glucose and inhibited cell growth in PDHA1^−/−^ cells (Fig. 6, *B*–*D*; Fig. S6*B*). Furthermore, PDK1 knockdown suppressed cell proliferation and PDHA1 phosphorylation (Fig. S6, *C* and *D*). Nevertheless, these data indicate that PDK inhibition impeded cell proliferation *via* PDHA1 activation.

It was noted that the concentration of DCA exceeding 10 mM, a level frequently employed in prior studies, showed severe off-target effects (Fig. 6*A*; Fig. S6*A*). Subsequently, we investigated two PDK inhibitors of AZD7545 and JX06, which were developed as more specific alternatives to DCA (38, 39). We observed that AZD7545 and JX06 significantly suppressed cellular growth, whereas the mechanism underlying this growth inhibition appeared complicated (Fig. S6, *E*–*H*). Interestingly, AZD7545 exhibited a more pronounced inhibitory effect on the growth of PDHA1 knockout cells compared with control cells (Fig. S6, *E* and *F*). And we found that AZD7545 effectively attenuated PDHA1 phosphorylation and unexpectedly increased PDK1 and PDK2 protein expression in both control and PDHA1 knockout cells (Fig. S6, *G* and *H*). Furthermore, treatment with 1 μM JX06 significantly inhibited cellular proliferation (Fig. S6, *G* and *H*), yet it did not exert any discernible influence on the phosphorylation of PDHA1 (Fig. S6, *I* and *J*), suggesting this inhibition may be independent of PDHc. These findings underscore the imperative need for the development of high targeting novel PDK inhibitors.

It was reported that YBX1 expression is required to maintain cancer cell proliferation (19, 40). Indeed, YBX1 knockdown suppressed cell growth rate, and PDK1 knockdown and DCA further enhanced this inhibitory efficiency (Fig. 6, *E* and *F*; Fig. S6, *K* and *L*), indicating that PDHA1 inactivation mitigates YBX1 knockdown–inhibited cell proliferation. To further explore this phenomenon *in vivo*, we subcutaneously implanted control vector and shRNA-mediated *Ybx1* knockdown 4T1 cells into mice and treated mice with DCA. The mice injected with *Ybx1* knockdown cells had smaller tumor volumes and weight compared with those receiving control vector cells (Fig. 6, *G*–*I*). In addition, DCA inhibited tumor growth rate more significantly in mice bearing *Ybx1* knockdown cells compared with control (Fig. 6, *G*–*J*). These data suggest that PDHA1 activation constrains the tumorigenic capacity of YBX1.

## Discussion

In the present study, we demonstrate that YBX1 functions as a key factor promoting pyruvate catabolism by activating PDHA1 activity through PDK inhibition. Given the importance of pyruvate oxidation in the regulation of body temperature within brown adipose tissues (41, 42), our observations may explain, at least partially, why chronic cold temperature–activated YBX1 promotes thermogenesis in adipocytes (43). Furthermore, YBX1 is highly expressed in different organs, including the brain, heart, muscle, and liver (44), and inactivation of YBX1 leads to embryonic development failure and cardiac dysfunction (14, 45). Similarly, a chronic reduction in PDH activity induces heart failure (46, 47, 48). Thus, the YBX1–PDH axis may provide a metabolic molecular basis for this process. YBX1 translationally enhances the expression of HIF1α (17), a well-known transcriptional activator of PDK1 (29, 49). However, the inactivation of YBX1 transcriptionally increases PDK1 expression in human cells, a phenomenon observed even in the context of reduced HIF1α expression. This observation implies a potential for mutual limitation of YBX1 and HIF1α activities on certain target genes under specific physiological conditions (50). This contradictory dual role of YBX1 in the HIF1α signaling pathway needed to be further investigated. In addition, YBX1 directly binds to the HRR of the human *PDK1* promoter, thereby repressing its transcription, a mode of regulation that is not observed in the mouse promoter, indicating that the 5`-CCTG-3′ sequence is required for YBX1 binding to the regulatory region of target genes (32). The difference in the molecular mechanisms governing YBX1–PDK1 regulation between human and mice is further supported by the evolvability of regulatory elements (51). Furthermore, we observed that PDK4 expression was inhibited by YBX1 both in human and mouse cells. Previous studies have indicated that YBX1 can activate PI3K–AKT pathway in various cancer cells (52, 53). Nevertheless, treatment with the AKT inhibitor, MK2206, did not alter PDK4 expression in either control or YBX1 knockdown cells, indicating that the regulation of PDK4 by YBX1 is independent of the canonical AKT-FOXO signaling pathway (24, 25, 26). The detailed mechanism needed to be further investigated.

PDKs and PDH phosphatases regulate PDHA1 activity by reversible phosphorylation (8). This regulation of PDHA1 activity has a great impact on NAD^+^ regeneration (10), hypoxia-adapted mitochondrial oxygen consumption (49), and the process of cellular senescence (9). YBX1 is a well-known oncogene, which plays a critical role in the regulation of gene expression involved in cancer progression. Loss of YBX1 expression significantly impedes cell proliferation in various cancer cells (16). Our previous study demonstrates that overexpression of YBX1 suppresses cell growth in some of the liver cancer cells (37), supporting that the effects of YBX1 on cell proliferation are complicated (54).

The transition from normal to malignant cells, prompted by oncogene activation, necessitates overcoming the oncogene-induced cell senescence (9, 55). Specifically, activation of PDH enhances the flux of pyruvate into the TCA cycle, leading to increased mitochondrial respiration and the accumulation of ROS, which collectively contribute to the establishment of the senescent phenotype (9). Our study demonstrates that YBX1-dependent PDHA1 activation suppresses cell proliferation and induces senescence in MCF-10A cells. Importantly, YBX1 is known to resist AKT activation–induced oncogenic transformation in chicken embryo fibroblasts (56, 57). Conversely, in malignant cells, where tumor suppressor pathways are often compromised and antioxidant mechanisms are upregulated, YBX1 expression supports tumor progression by promoting cell proliferation and survival. Interestingly, our previous research has revealed that YBX1 exhibits distinct interactions with MPCs (MPC1/2) depending on the cellular context (20). In tumor cells, YBX1 localizes to the mitochondrial intermembrane space and binds to MPC1/2, thereby inhibiting mitochondrial pyruvate uptake. Conversely, in MEF cells, YBX1 shows minimal mitochondrial localization and does not interact with MPC1/2, suggesting a cell type–specific regulatory mechanism. This differential behavior underscores the multifaceted roles of YBX1 in cellular metabolism and highlights the importance of cellular context in determining its function.

Whether YBX1–PDHA1 participates in regulating cancer cell initiation is unknown and deserves further investigation. Nevertheless, these findings provide a novel perspective on the mechanism of YBX1 in regulating PDHA1 activity, which in turn influences pyruvate metabolism and cell fate.

## Experimental procedures

### Cell culture, transfection, and lentivirus production

*Ybx1*^−/−^ MEFs were generated as previously described (20). MEFs (passage number ranges are P5-10), HEK293T (passage number ranges are P5-20), MDA-MB-231 (passage number ranges are P5-20), and HBE (passage number ranges are P5-20) cells were cultured in DMEM with 10% fetal bovine serum (FBS). MCF-10A (passage number ranges are P5-10) cells were cultured in DMEM with 5% FBS, 20 ng/ml epidermal growth factor, 10 μg/ml insulin, 0.5 μg/ml hydrocortisone, and 100 ng/ml cholera toxin. HEK293T cells were transfected using polyethylenimine. Transfection mass ratio of plasmids to polyethylenimine was 1:3. Lentivirus production was performed using two systems. For the two-plasmid packaging system, psPAX2 and pVSVg together with targeting plasmids were cotransfected into HEK293T cells for 48 h to harvest the supernatant of lentivirus. For the three-plasmid packaging system, pMDLg, pVSVg, and pREV were used. Targeting cells were incubated with the medium mixed with the indicated supernatant of lentivirus for 24 h. Next, the cells were kept in the normal culture medium and used for further treatment.

### *In vivo* animal studies

Ybx1^*fl/fl*^ and Alb-cre mice were generated and obtained from GemPharmatech. Liver-specific Ybx1 knockout mice were generated by crossing Ybx1^*flox/flox*^ and Alb-cre strain. The male 8-week-old Ybx1^*cKO*^ and WT littermates were used to metabolites and protein detection after fasted and refed treatment as described. Briefly, mice were pretrained with 12 h fasting and 12 h refeeding daily for 3 days before the experiment. On the last day, the refed mice were free to standard diet for 8 h, and the other mice continued to fast for 8 h followed by further treatment. Female BALB/c mice (6–8 weeks old) were obtained from the Dalian Medical University and maintained under specific pathogen-free conditions. For tumor growth assay, 4T1 cells (1 × 10^5^ per mice) in 200 μl of cells suspension (mixed with Matrigel at a 1:1 ratio) were injected subcutaneously. DCA (0.75 g/l) was added to the drinking water as previously described (27). The tumors were removed, photographed, and weighed 25 days after injection. All institutional and national guidelines for the care and use of laboratory animals were followed. All animal care and experimental procedures were approved by the Research Ethics Committee of Dalian Institute of Chemical Physics, Chinese Academy of Sciences.

### Phosphoproteomic sample preparation and database searching

Sample preparation and phosphopeptide enrichment were performed as previously described (58, 59). Briefly, cells were washed with ice-cold PBS twice and extracted by an ice-cold lysis buffer (50 mM Tris–HCl, 8 M urea, 1% Triton X-100, and phosphatase and protease inhibitor cocktail) in the ice bath. After sonication for 10 min at 400 W, cells were spun down at 12,000*g* for 30 min. The supernatant was precipitated in ice-cold acetone/ethanol/acetic acid (50/50/0.1, v/v/v). Next, the pellet was redissolved in buffer containing 8 M urea and 100 mM triethylamine bicarbonate (pH 8.0), and the protein concentration was determined using the bicinchoninic acid assay. Appropriate proteins were reduced by 5 mM triethylamine bicarbonate and alkylated by 10 mM iodoacetamide, followed by diluting to 1 M urea by 100 mM triethylamine bicarbonate buffer (pH 8.2). Protein lysate was digested with trypsin/protein (1:20, w/w) overnight at 37 °C. After digestion, 1% TFA was added to stop the reaction, and the peptide mixture was kept at −80 °C until enrichment. Phosphopeptide enrichment was performed totally according to a previous study (59). MaxQuant (Cox Laboratory, Max Planck Institute of Biochemistry) (60) software suite (version 1.6.4.0) was used for mass spectrometry data searching, and data analysis was supported by the robust statistical tool Perseus (Cox Laboratory, Max Planck Institute of Biochemistry) (61) software (version 1.5.8.5) using default settings, including *t* test. Database searching and analysis were performed as previously described (58). Pathway information was obtained from the Kyoto Encyclopedia of Genes and Genomes. The mass spectrometry–based phosphoproteomics data are linked in URL: https://doi.org/10.57760/sciencedb.28170.

### Western blot

Cells were harvested and extracted proteins using ice-cold lysis buffer (150 mM NaCl, 1% Triton X-100, 1 mM EDTA, 1 mM EGTA, 2.5 mM sodium pyrophosphate, 1 mM β-glycerolphosphate, 20 mM Tris–HCl, pH 7.5, with protease and phosphatase inhibitor cocktail). After the addition of the lysis buffer, cell lysates were subjected to brief sonication on ice (10–15 cycles of 2 s each) to avoid protein denaturation. Subsequently, the lysates were centrifuged at 10,000g for 10 min at 4 °C. Protein concentrations were determined using a Bradford assay. The supernatants were then denatured with 5× SDS sample loading buffer. The samples were analyzed by SDS-PAGE. For immunoblotting, the proteins were transferred from the gel to a 0.45-μm polyvinylidene fluoride membrane at 100 V for 1 h using a transfer buffer. The membrane was subsequently blocked with 5% nonfat dry milk and incubated with the respective primary antibodies.

### CRISPR–Cas9 knockout of YBX1 and PDHA1

The YBX1 knockout MDA-MB-231 cell line was acquired as previously reported (20). Single guide RNA targeting sequences were designed using the CHOPCHOP online platform. The target sequences for PDHA1 were 5′-GGATATCCTGTGCGTCCGAG-3′ and 5′-AAGCTTCGAATATCTGGCCC-3′. The targeting sequences were cloned into LentiCRISPR v2 vector. After infection, both MDA-MB-231 and HEK293T cells were selected with puromycin (1 μg/ml) for 48 h. Subsequently, the cells were allowed to recover for another 24 h in the absence of puromycin. Cells were then seeded into 96-well plates at a density of approximately 50 cells per well to isolate single-cell colonies. Upon the formation of colonies, knockout of the target genes was confirmed by Western blot analysis.

### PDH enzymatic activity analysis

PDH activity was evaluated with the PDH Activity Colorimetric Assay Kit according to the manufacturer’s instructions. In summary, approximately 1 × 10^6^ cells were lysed in 100 μl of ice-cold PDH assay buffer for 10 min. The lysates were centrifuged at 10,000*g* at 4 °C for 5 min. A 50-μl aliquot of the cleared supernatant was transferred to a 96-well plate containing 50 μl reaction mix, which included substrate and developer. The absorbance was measured at 450 nm using a plate reader (Cytation5; BioTek).

### Cell growth measurement

The assessment of cell proliferation was conducted using the Cell Counting Kit-8 (CCK-8) according to the manufacturer’s instructions. Briefly, cells were seeded at a density of 1000 to 2000 cells per well in a 96-well plate. At specified time points (24 h apart), the cells were treated with 100 μl of culture medium supplemented with 10% CCK-8 assay solution. Following a 2-h incubation period, the absorbance was measured at 450 nm using a microplate reader (Cytation5).

For the growth assay involving various concentrations of DCA, AZD7545, and JX06, 1000 cells were initially plated in each well of a 96-well plate. After 24 h of attachment, the initial cell numbers were determined using the CCK-8 assay. Subsequently, the medium in the remaining wells was replaced with fresh medium containing different concentrations of DCA, AZD7545, or JX06. Following an incubation period of 48 to 72 h, cell viability was assessed using the CCK-8 assay to determine the number of viable cells.

### Colony formation assay

Cells were seeded into a 6-well plate at a density of 200 cells per well. Upon visible colonies had formed, the cells were fixed and stained with crystal violet buffer (0.1% crystal violet in a mixture of methanol/water, v/v, 20/80). The plates were then washed with PBS three times to remove excess stain. Finally, the plates were photographed, and the number of colonies were quantified using ImageJ software (National Institutes of Health).

### SA-β-gal activity analysis

The assessment of SA β-gal activity was conducted using the Senescence β-Galactosidase Staining Kit according to the manufacturer’s instructions. Briefly, MCF-10A cells were seeded into a 24-well plate at a density of 20,000 cells per well. Until cells reached to a confluence of 70% to 80%, the plates were washed with PBS once, followed by fixation with provided fixative buffer. Postfixation, the wells were washed with PBS three times to clear residual fixative. The cells were then incubated with staining solution containing X-Gal overnight at 37 °C. Finally, the wells were photographed using a microscope equipped with camera (DMi1; Leica), and β-gal activity was quantitively analyzed using the ImageJ software.

### Cellular ROS measurements

The assessment of cellular ROS was conducted using Reactive Oxygen Species Assay Kit according to the manufacturer’s instructions. Briefly, cells were seeded in the black opaque 96-well plate at a density of 10,000 per well. After 24 h, the cells were incubated with provided ROS probe in DMEM without FBS for 30 min. The cells were washed with FBS-free DMEM three times to remove residue probe. Finally, the levels of ROS were interpreted as the fluorescence excitation/emission wavelengths at 488/525 nm using a microplate reader (Cytation5).

### 5-Ethynyl-2′-deoxyuridine staining

Cell proliferation was assayed according to the protocol of the BeyoClick EdU-594 kit (Beyotime). Briefly, MCF-10A cells were seeded in the 24-well plate at a density of 150,000 per well. After 24 h, the cells were incubated with 10 μM 5-ethynyl-2′-deoxyuridine working solution for 5 h. Afterward, cells were fixed with 4% paraformaldehyde for 15 min and 0.3% Triton X-100 for 10 min. Following being covered with reaction buffer and dyed by 4′,6-diamidino-2-phenylindole, the plates were pictured and quantified by a microplate reader (Cytation5).

### Luciferase reporter assay

*PDK1* promoter region (from −706 to +817) was cloned into the pGL4.16 vector (Promega). HEK293T cells were plated in a 24-well plate and allowed to adherent for 24 h. Subsequently, cells were transfected with the indicated plasmids. After 48 h post-transfection, the luciferase activity was measured using Luciferase Reporter Gene Assay Kit as per the manufacturer’s specification. The luminescent signal was quantified by a plate reader (Cytation5).

### Hypoxia treatment

Cells were cultured in a chamber (Nuvair) flushed with a gas mixture of 1% O_2_, 5% CO_2_, and 94% N_2_ at 37 °C for the indicated period.

### Quantitative RT–PCR and mRNA stability analysis

Total RNA was extracted from cells using the RNAiso Plus reagent. One microgram of the isolated RNA was subjected to reverse transcription using RT reagent Kit with Genomic DNA Eraser. Quantitative qRT–PCR was performed with the TB Green Fast qPCR Mix on a CFX96 instrument (Bio-Rad). For the analysis of *PDK1* mRNA stability, cells were treated with 5 μg/ml actinomycin D and harvested at indicated time points. Total RNA and quantitative RT–PCR were performed as described previously. Primers for target genes are provided in Table S1. Data analysis was performed using the ΔC_T_ method. The results are represented as mean ± SD of three or four biological replicates.

### Chromatin immunoprecipitation assay

MDA-MB-231 and 4T1 cells were seeded in 10 cm plates at a density of 5 × 10^6^ and allowed to adherent for 24 h. Chromatin extraction and immunoprecipitation were performed with a chromatin immunoprecipitation assay kit according to the manufacturer’s instructions. Antibodies used in the assay were anti-YBX1 (1:100 dilution; CST, #9744) and antinormal rabbit IgG (provided by chromatin immunoprecipitation assay kit). Immunoprecipitated target DNA was quantified using quantitative PCR analysis as described previously. The primers are provided in Table S1.

### OCR measurement

MEF cells were plated in 24-well plate at a density of 2.5 × 10^4^ cells per well (Agilent Technologies). The OCR measurement was conducted using XFe24 extracellular flux analyzer (Agilent Technologies) according to the manufacturer’s instructions. The XF DMEM measurement medium was supplemented with 2 mM glutamine, 10 mM glucose, and 2 mM pyruvate. The compounds used in the assay were oligomycin (2 μM), FCCP (0.75 μM), and rotenone/antimycin A (0.5 μM/0.5 μM).

### GC–MS-based metabolite analysis and stable isotope tracing *in vivo* and *in vitro*

For untargeted metabolite measurement, cells were changed to the fresh medium and incubated for 24 h. After incubation, cells were washed with ice-cold wash buffer (0.9% NaCl) two times and frozen with liquid nitrogen. Extracellular metabolites were tested by using 20 μl of incubated medium. [U-^13^C]cc glucose was purchased from Cambridge Isotope Laboratories. For the cultured cell tracing experiment, [U-^13^C] glucose (10 mM) was substituted for the relative metabolite in DMEM (no glucose, no glutamine, no pyruvate, and no phenol red) containing 10% dialyzed FBS, and the rest of the metabolites were unlabeled. After the tracing experiment, cells were washed with cold wash buffer (0.9% NaCl) two times and frozen in liquid nitrogen.

For *in vivo* [U-^13^C] glucose tracing, fasted or fasted-refed Ybx1 WT and Ybx1^*cKO*^ mice were intraperitoneally injected with [U-^13^C] glucose (2 g/kg) for 15 min (41). Subsequently, the mice were sacrificed, and their liver tissues were rapidly frozen in liquid nitrogen. Sample preparation and metabolomics analysis methods were conducted as previously described (62). Briefly, cells were extracted by an ice-cold lysis buffer (methanol/water, 4/1, v/v, 1 μg/ml tridecanoic acid, and 1 μg/ml norvaline). After addition of the lysis buffer, cell lysates were subjected to brief sonication on ice (5–10 cycles of 2 s each), followed by centrifugation at 12,000*g* for 10 min at 4 °C. The supernatants were then transferred to a new tube and dried out using a vacuum freeze-drying machine. The lyophilized metabolites were derivatized by methoxyamine hydrochloride (20 mg/ml), followed by derivatization with 2,2,2-trifluoro-*N*-methyl-*N*-trimethylsilylacetamide for untargeted metabolite measurement or *N*-tertbutyldimethylsilyl-*N*-methyltrifluoroacetamide for metabolic flux analysis. Metabolite identification and data analysis was conducted as previously described (63). Natural isotope abundance was corrected using IsoCor, v.2.0 (Metabolic Flux Analysis group (MetaToul), INRAE, France) (64).

### Statistical analysis

Data are processed using GraphPad Prism, version 5.0 (GraphPad Software) and presented as mean ± SD. Two-tailed unpaired Student’s *t* test (normal distribution) was used to assess the significance between two groups. *p* < 0.05 was considered statistically significant.

## Data availability

The article includes all study data and supporting information.

## Supporting information

This article contains supporting information.

## Conflict of interest

The authors declare that they have no conflicts of interest with the contents of this article.
