# Supplementary material for: The pyruvate dehydrogenase complex in concert with the DNA/RNA-binding protein YBX1 regulates cell senescence and tumorigenesis
Source: J Biol Chem. 2025 Aug 12;301(9):110585. doi: 10.1016/j.jbc.2025.110585 (PMC12446528; doi:10.1016/j.jbc.2025.110585)
Supplement: Table S1 [file mmc1.docx]

| Species | Gene | Primer | 5' to 3' |
| --- | --- | --- | --- |
|  |  |  |  |
| Human | PDK1 | F | TCACCAGGACAGCCAATACA |
|  |  | R | TCCTCGGTCACTCATCTTCAC |
|  | PDK2 | F | AGAGCTGGTATGTCCAGAGC |
|  |  | R | GCCGTAGGTGTCCTTGTACT |
|  | PDK3 | F | CCCTTTGGCTGGATTTGGTT |
|  |  | R | CAGGCGTGGTCTTGTAATGG |
|  | PDK4 | F | CATACTCCACTGCACCAACG |
|  |  | R | CGAGAAATTGGCAAGCCGTA |
|  | PDP1 | F | CGTCGGGAAGAATCGTTTGG |
|  |  | R | GGCATGGCATCAGAGAACAG |
|  | PDP2 | F | GGTAGACGCTTATACTCCAGGT |
|  |  | R | CACATGGGGAACTGTTTAGGG |
|  | PDHA1 | F | TTACCGTTACCACGGACACA |
|  |  | R | TGGCAAGATTGCTGTTCACC |
|  | GLUT1 | F | TCTGGCATCAACGCTGTCTTC |
|  |  | R | CGATACCGGAGCCAATGGT |
|  | GAPDH | F | GACAGTCAGCGCATCTTCT |
|  |  | R | GCGCCCAATACGACCAATC |
|  |  |  |  |
| Species | Gene | Primer | 5' to 3' |
| Mouse | PDK1 | F | GCGAAATCACCAGGACAGAC |
|  |  | R | CCCGGTCACTCATCTTCACA |
|  | PDK2 | F | GCAGAGCTGGTATGTCCAGA |
|  |  | R | CTTAGAGTCCGGTGGTCCTC |
|  | PDK3 | F | AACCGCATTTCTTTCCGCAT |
|  |  | R | TTCGGATGTGCAGGGTTAGT |
|  | PDK4 | F | CCGCATTTCTACTCGGATGC |
|  |  | R | AGGCATCTTGGACTACTGCT |
|  | PDP1 | F | CAGGAGAATGTGTGTGTGTCC |
|  |  | R | CTGGCATGGCATCAGAGAAC |
|  | PDP2 | F | GACGAGGATACGAGGCTGAA |
|  |  | R | GTATAGGCGTCTCCCACCTC |
|  | PDHA1 | F | TGCAGAGCTAACAGGACGAA |
|  |  | R | TTGGCGTACATGTGCATTGA |
|  | GAPDH | F | GGACTTCGGGTCAGTGAATGC |
|  |  | R | TCCTGAGAAGATTGTCGGGGA |

**Table S1. QRT-PCR primer sequences were used in this study.**

| Name | | Primer | 5' to 3' |
| --- | --- | --- | --- |
| PDK1 promoter | amplicon 1 | F | GAGGTCTCCTGTCTCCCTGT |
|  |  | R | CTTGACAAGGTTGAGGACGC |
|  | amplicon 2 | F | GCCTCTTAGTGTCCCCAGTC |
|  |  | R | GCCTCCCGAGGAAGGAGAT |
