# Supplementary material for: The pyruvate dehydrogenase complex in concert with the DNA/RNA-binding protein YBX1 regulates cell senescence and tumorigenesis
Source: J Biol Chem. 2025 Aug 12;301(9):110585. doi: 10.1016/j.jbc.2025.110585 (PMC12446528; doi:10.1016/j.jbc.2025.110585)
Supplement: Table S2 [file mmc2.docx]

**Table S2. The list of key reagent and resource information.**

| REAGENT or RESOURCE | RESOURCE | IDENTIFIER |
| --- | --- | --- |
| **Antibodies (WB dilution ratio)** | | |
| Rabbit polyclonal anti-phospho-PDH-s232 WB: 1:1000 | Sigma | Cat# AP1063; RRID:AB_10616070 |
| Rabbit polyclonal anti-phospho-PDH-s293 WB: 1:5000 | Sigma | Cat# AP1062; RRID:AB_10616069 |
| Rabbit polyclonal anti-phospho-PDH-s300 WB: 1:2000 | Sigma | Cat# AP1064; RRID:AB_10618090 |
| Rabbit polyclonal anti-YBX1 WB: 1:1000 | CST | Cat# 9744; RRID:AB_11178953 |
| Rabbit polyclonal anti-PDK1 WB: 1:1000 | CST | Cat# 3820; RRID:AB_1904078 |
| Rabbit polyclonal anti-P-S6 WB: 1:1000 | CST | Cat# 2211; RRID:AB_331679 |
| Rabbit polyclonal anti-Phospho-AKT(Thr308) WB: 1:1000 | CST | Cat# 4056; RRID:AB_331163 |
| Rabbit polyclonal anti-FOXO1 WB: 1:1000 | CST | Cat# 2880; RRID:AB_2106495 |
| Rabbit polyclonal anti-Phospho-FOXO1(Ser256) WB: 1:1000 | CST | Cat# 9461; RRID:AB_329831 |
| Rabbit polyclonal anti-ERK1/2 WB: 1:1000 | CST | Cat# 4695;  RRID:AB_390779 |
| Rabbit polyclonal anti-Phospho-ERK1/2(Thr202/204) WB: 1:1000 | CST | Cat# 4370;  RRID:AB_2315112 |
| Rabbit polyclonal anti-PDK2 WB: 1:1000 | Proteintech | Cat# 15647-1-AP; RRID:AB_2268006 |
| Rabbit polyclonal anti-PDK4 WB: 1:1000 | Proteintech | Cat# 12949-1-AP; RRID:AB_2161499 |
| Rabbit polyclonal anti-P16 WB: 1:1000 | Proteintech | Cat# 10883-1-AP;  RRID:AB_2919007 |
| Mouse monoclonal anti-GAPDH WB: 1:10000 | Proteintech | Cat# 60004-1-Ig; RRID:AB_2107436 |
| Rabbit polyclonal anti-HIF1αWB: 1:1000 | GeneTex | Cat# GTX127309; RRID:AB_2616089 |
| Mouse monoclonal anti-HA tag WB: 1:1000 | Santa cruz | Cat# sc7392; RRID:AB_627809 |
| Mouse monoclonal anti-Vinculin WB: 1:2000 | Santa cruz | Cat# sc73614; RRID:AB_1131294 |
| Mouse monoclonal anti-PDHA1 WB: 1:1000 | Santa cruz | Cat# sc377092; RRID:AB_2716767 |
| Mouse monoclonal anti-AKT1 WB: 1:1000 | Santa cruz | Cat# sc-5298;  RRID:AB_626658 |
| Mouse monoclonal anti-S6 WB: 1:1000 | Santa cruz | Cat# sc-74459; RRID:AB_1129205 |
| HRP-linked anti-rabbit IgG WB: 1:20000 | Jackson ImmunoResearch | Cat# 111-035-003; RRID: AB_2313567 |
| HRP-linked anti-mouse IgG WB: 1:20000 | Jackson ImmunoResearch | Cat# 115-035-003; RRID: AB_10015289 |
| **Chemicals, Peptides, and Recombinant Proteins** | | |
| ECL | Advansta | Cat# K-12045-D50 |
| ^13^C-Glucose | Cambridge Isotope Laboratories | Cat# CLM-1396 |
| Dilyzed FBS | Serana | Cat# S-FBS-US-065 |
| Methoxyamine hydrochloride | Sigma | Cat# 89803 |
| MSTFA | Sigma | Cat# 69479 |
| MTBSTFA | Sigma | Cat# M-108 |
| DMEM (no glucose, no glutamine, no pyruvate, no phenol-red) | Sigma | Cat# D5030 |
| Glutamine | Sigma | Cat# G3126 |
| Sodium pyruvate | Sigma | Cat# P4562 |
| D-glucose | Gbico | Cat# 15023-021 |
| Polybrene | Sigma | Cat# H9268 |
| Puromycin | InvivoGen | Cat# ant-pr-1 |
| Blasticidin | InvivoGen | Cat# ant-bl-1 |
| PEI | Polysciences | Cat# 24765 |
| Protease inhibitor cocktail | Bimake | Cat# 14002 |
| Phosphatase inhibitor cocktail | Bimake | Cat# 15002 |
| DCA | Santa Cruz Biotechnology | Cat# sc-214877 |
| Oligomycin | Abcam | Cat# ab141829 |
| FCCP | MCE | Cat# HY-100410 |
| Antimycin A | MCE | Cat# HY-105755 |
| Actinomycin D | Apexbio | Cat# A4448 |
| Rotenone | MCE | Cat# HY-B1756 |
| Cholera toxin | Sigma | Cat# C-8052 |
| EGF | Beyotime | Cat# P5552 |
| Insulin | Sigma | Cat# 91077C |
| Hydrocortisone | Selleck | Cat# S5501 |
| RNAiso Plus reagent | Takara | Cat# 9109 |
| RT reagent kit with gDNA Eraser | Takara | Cat# RR047A |
| TB Green Fast qPCR Mix | Takara | Cat# RR430S |
| Pyruvate dehydrogenase (PDH) activity colorimetric assay kit | BioVision | Cat# K679 |
| Luciferase reporter gene assay kit | Beyotime | Cat# RG089S |
| BCA assay kit | TIANGEN BIOTECH | Cat# PA115 |
| Cell counting Kit 8 (CCK-8) | MCE | Cat# HY-K0301 |
| **Cell lines** | | |
| HEK293T | ATCC | ACS-4500 |
| MDA-MB-231 | ATCC | CRM-HTB-26 |
| MCF-10A | ATCC | CRL-10137 |
| HBE | ATCC | CRL-2741 |
| 4T1 | ATCC | CRL-2539 |
| *Ybx1l*^fl/fl^ MEFs | This paper | N/A |
| *YBX1*^-/-^ MDA-MB-231 | This paper | N/A |
| *PDHA1*^-/-^ MDA-MB-231 | This paper | N/A |
| *PDHA1*^-/-^ HEK-293T | This paper | N/A |
| **Organism/Strains** | | |
| Female BALB/c mice | Beijing Vital River Laboratory Animal Technology Co., Ltd (Beijing, China) | N/A |
| C57BL/6JGpt-*Ybx1*^fl/fl^ | GemPharmatech Co. (Nanjing, China) | N/A |
| C57BL/6JGpt-Alb^cre^ | GemPharmatech Co. (Nanjing, China) | N/A |
| WT *Ybx1* mouse: C57BL/6JGpt-*Ybx1l*^f/fl^  *Ybx1* Liver KO mouse: C57BL/6JGpt-*Ybx1l*^f/fl^ + Alb^cre^ | This paper | N/A |
| **Bacterial and Virus Strains** | | |
| E.coli. Stbl3 competent cells | Transgene | Cat# CD201 |
| E.coli. DH5α competent cells | Transgene | Cat# CD521 |

| **Oligonucleotides** | | |
| --- | --- | --- |
| pLKO.1-puro-shYBX1-1:  CCAGTTCAAGGCAGTAAATAT | Sigma | TRCN0000315307 |
| pLKO.1-puro-shYBX1-2:  AGCAGACCGTAACCATTATAG | Sigma | TRCN0000315309 |
| pLKO.1-bsd/puro-shPDK1: GCTCTGTCAACAGACTCAATA | This paper | N/A |
| pLKO.1-puro-shPDK1-2:  CCAGGGTGTGATTGAATACAA | This paper | N/A |
| pLKO.1-puro-shPDK1-3: CATCCGTTCAATTGGTACAAA | This paper | N/A |
| pLKO.1-puro-shPDK1-4: CGTGAATATGTTGAAGTAGAA | This paper | N/A |
| pLKO.1-puro-shPDK1-5: GAAGTAGAAGTCTACCATATT | This paper | N/A |
| sgRNA targeting sequence: *hYBX1*:  GTTCCCAAAACCTTCGTTGC | http://chopchop.cbu.uib.no/ | N/A |
| sgRNA targeting sequence: *hYBX1*:  GTTTTCTTTTCCAGCAACGA | http://chopchop.cbu.uib.no/ | N/A |
| sgRNA targeting sequence: *hPDHA1*:  GGATATCCTGTGCGTCCGAG | http://chopchop.cbu.uib.no/ | N/A |
| sgRNA targeting sequence: *hPDHA1*:  AAGCTTCGAATATCTGGCCC | http://chopchop.cbu.uib.no/ | N/A |
| Primers for qRT-PCR analysis, see Table S1 | This paper | N/A |
| Primers for ChIP analysis, see Table S1 | This paper | N/A |
| **Recombinant DNA** | | |
| pLKO.1-puro | Dr. Zhang CS | N/A |
| pLKO.1-bsd | This paper | N/A |
| pBOBI vector | Dr. Zhang CS | N/A |
| lentiCRISPRv2 | Dr. Feng Zhang | Addgene 52961 |
| Three-plasmid lentivirus packaging system | Dr. Zhang CS | N/A |
| Two-plasmid lentivirus packaging system | This paper | N/A |
| HA-YBX1 pBOBI | This paper | N/A |
| Cre pCDH | Dr. Zhang CS | N/A |
| PDK1 pBOBI | This paper | N/A |
| BRAF-V600E PCW | This paper | N/A |
| hPDK1 promoter pGL4.16 | This paper | N/A |

| **Software and Algorithms** | | |
| --- | --- | --- |
| ImageJ | NIH | https://imagej.nih.gov/ij/ |
| GraphPad Prism | GraphPad software | N/A |
| Excel | Microsoft | N/A |
