# Supplementary figures and images for: The pyruvate dehydrogenase complex in concert with the DNA/RNA-binding protein YBX1 regulates cell senescence and tumorigenesis
Source: J Biol Chem. 2025 Aug 12;301(9):110585. doi: 10.1016/j.jbc.2025.110585 (PMC12446528; doi:10.1016/j.jbc.2025.110585)

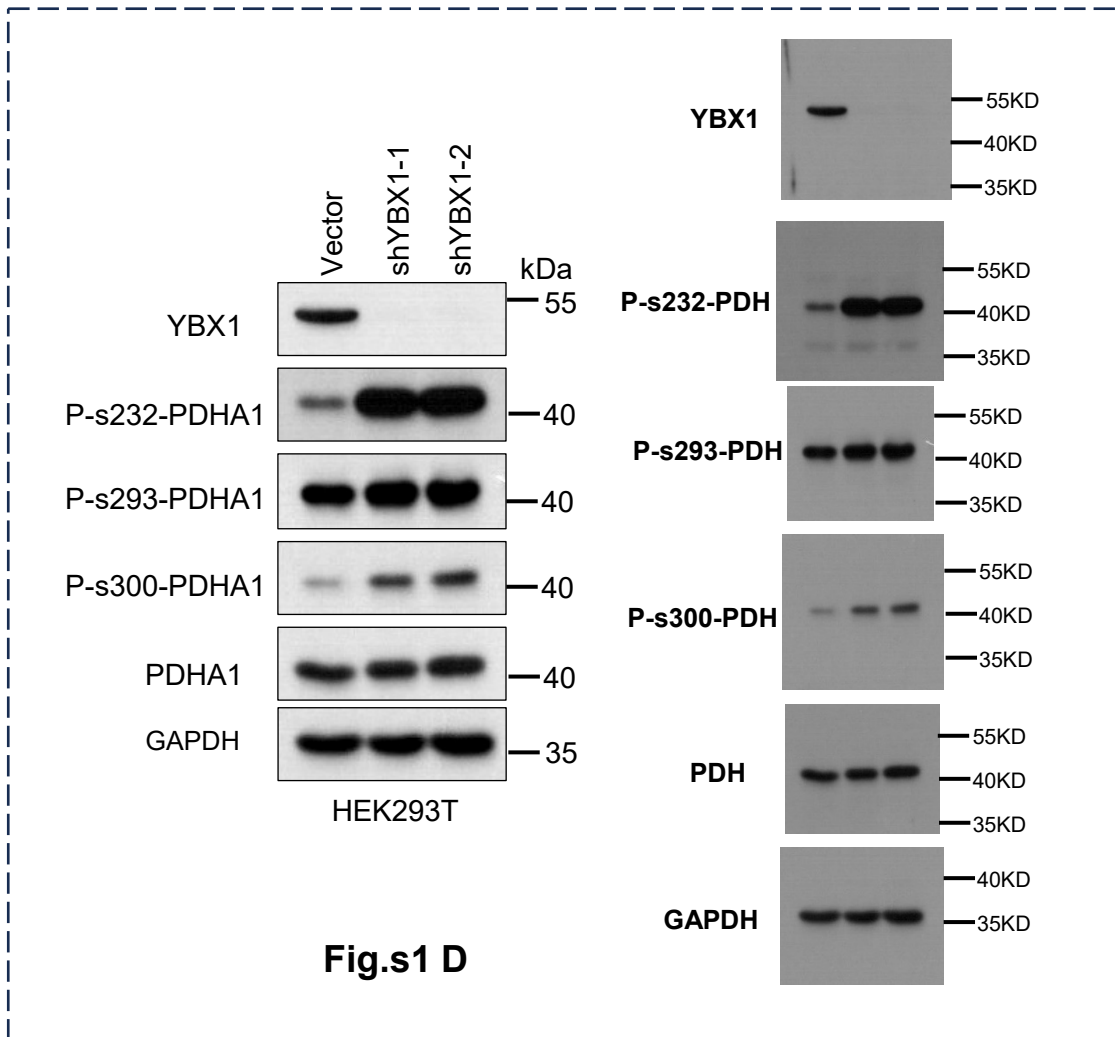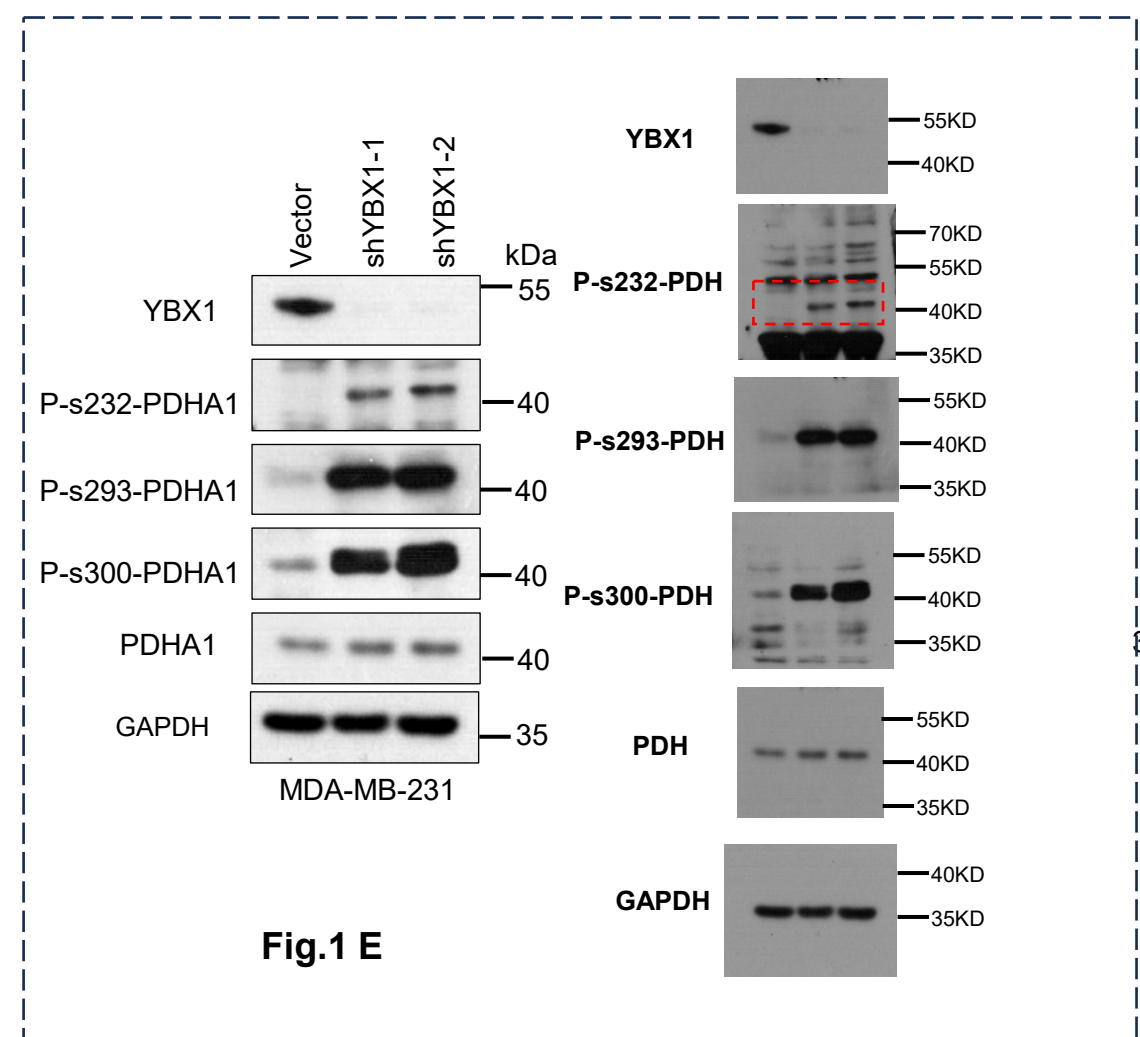

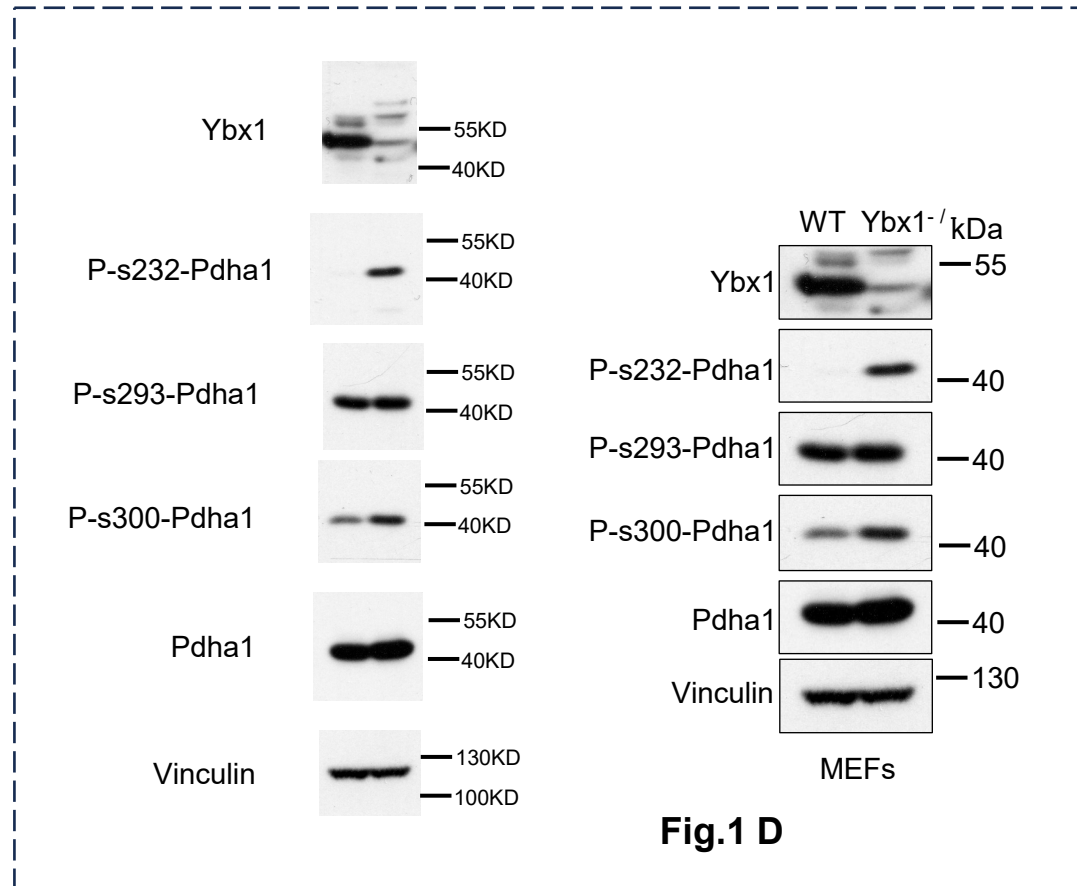

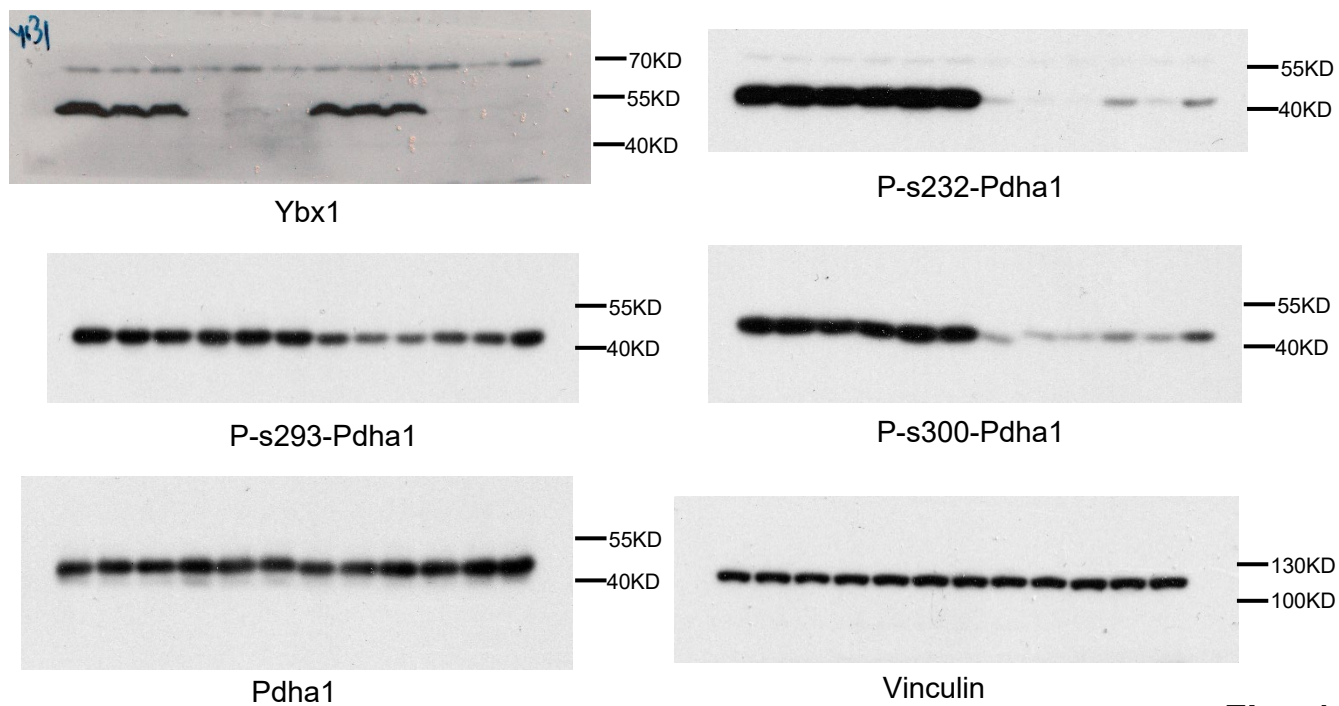

**Fig.s1 F**

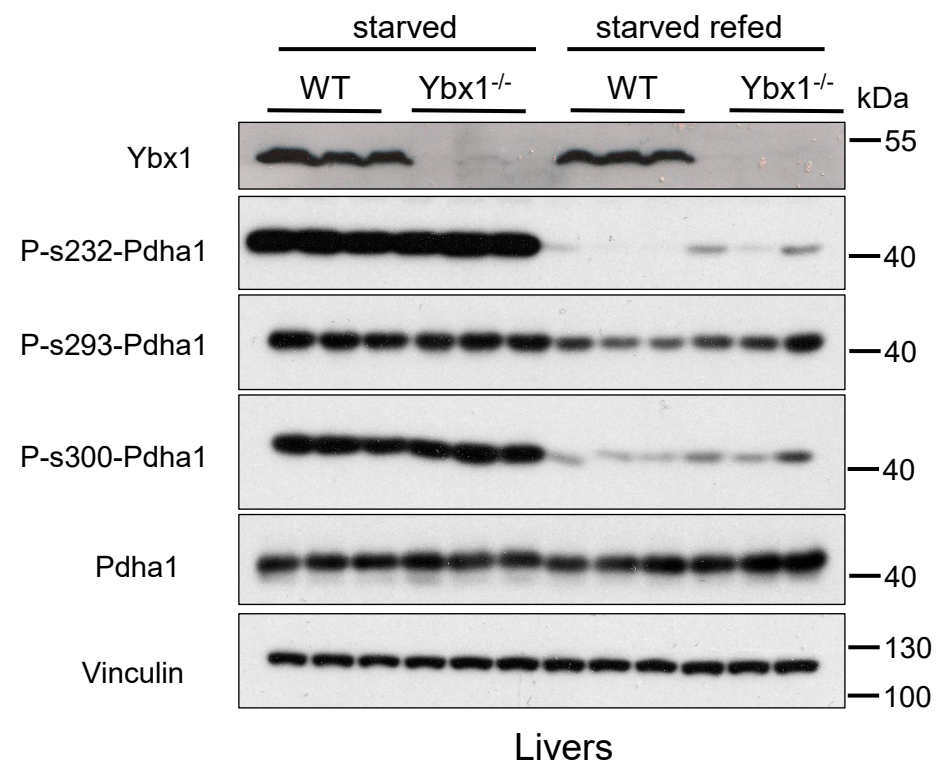

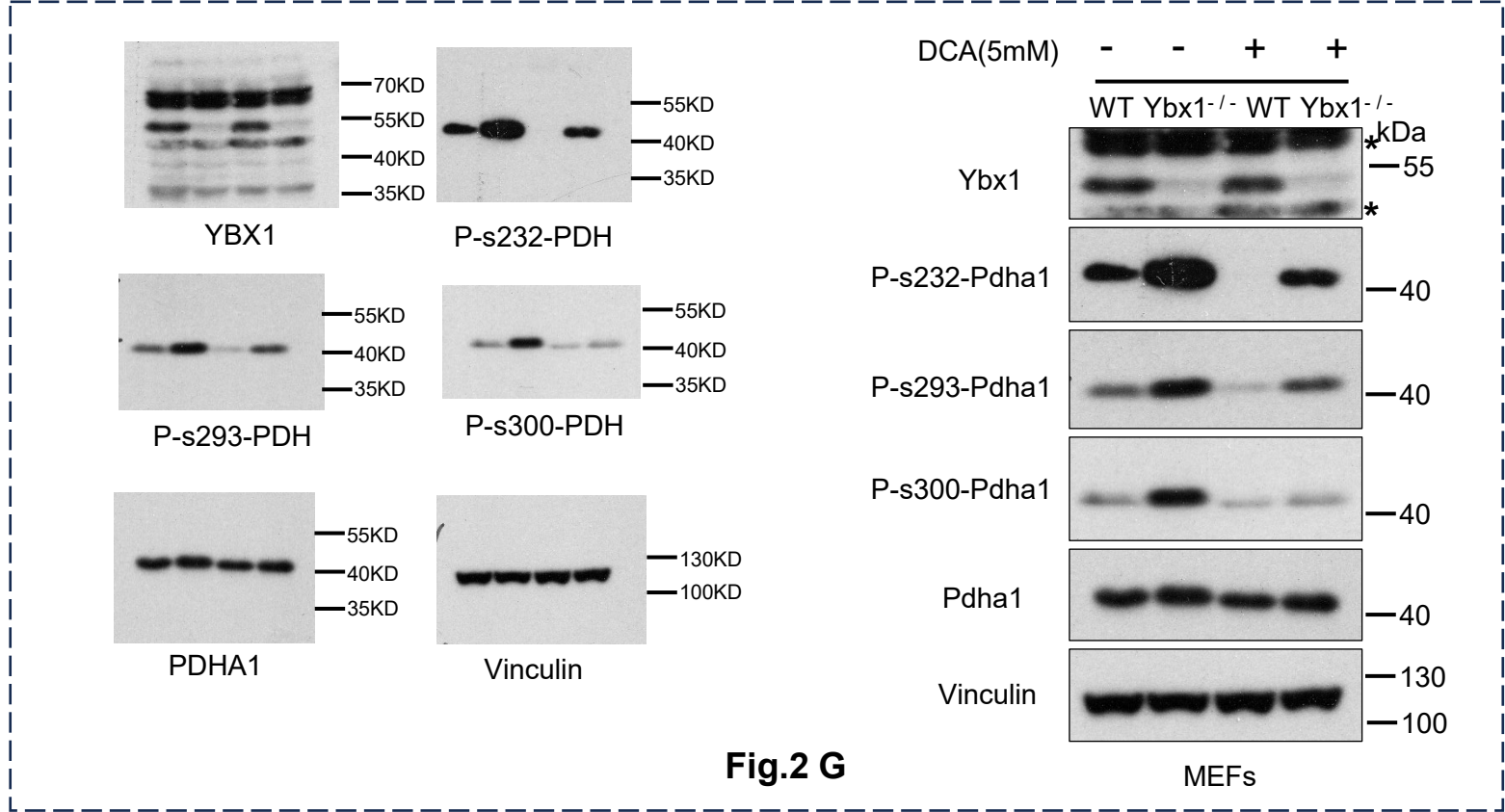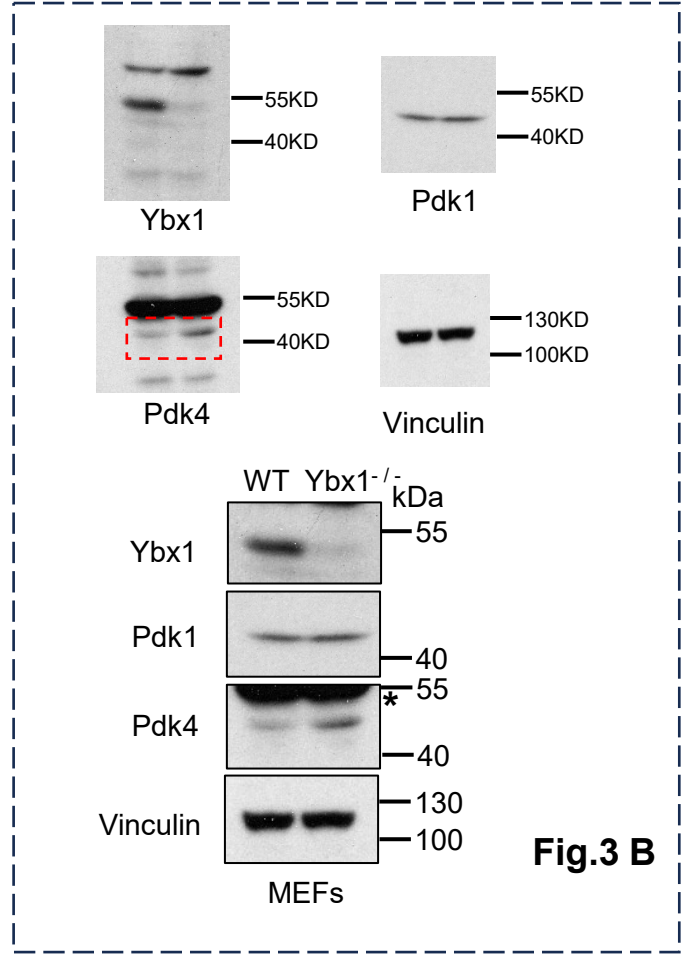

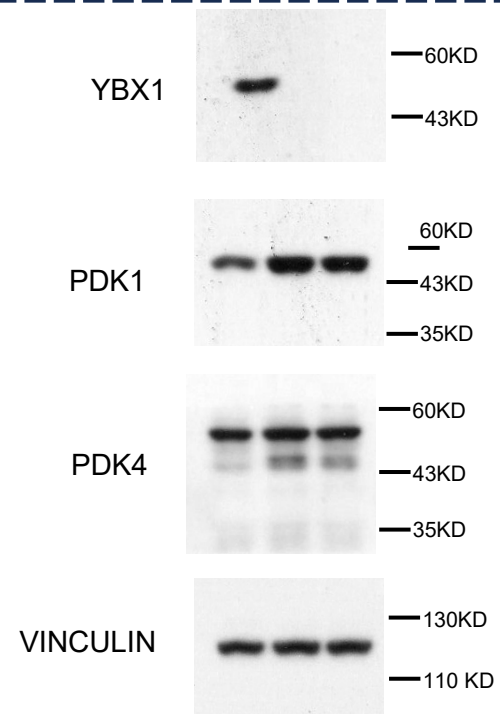

**Fig.3 E**

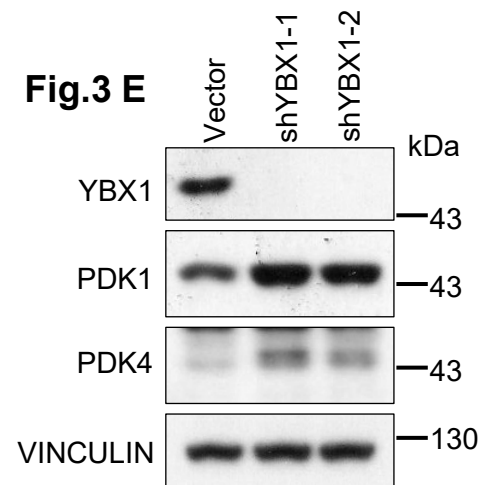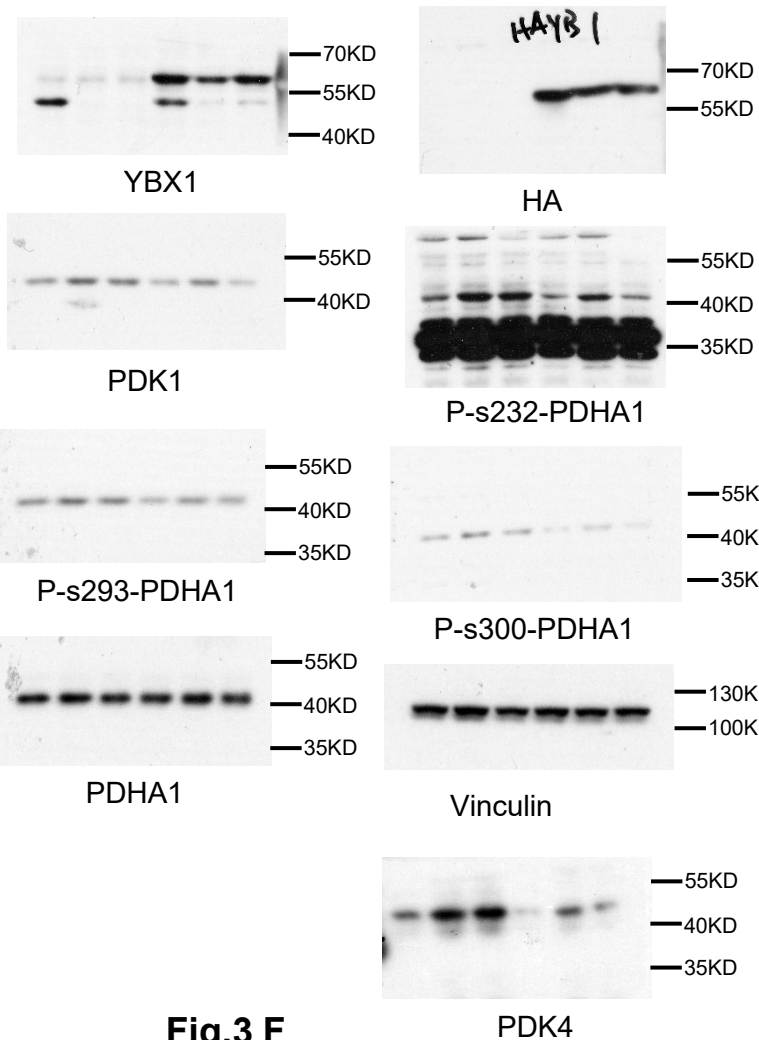

**Fig.3 F**

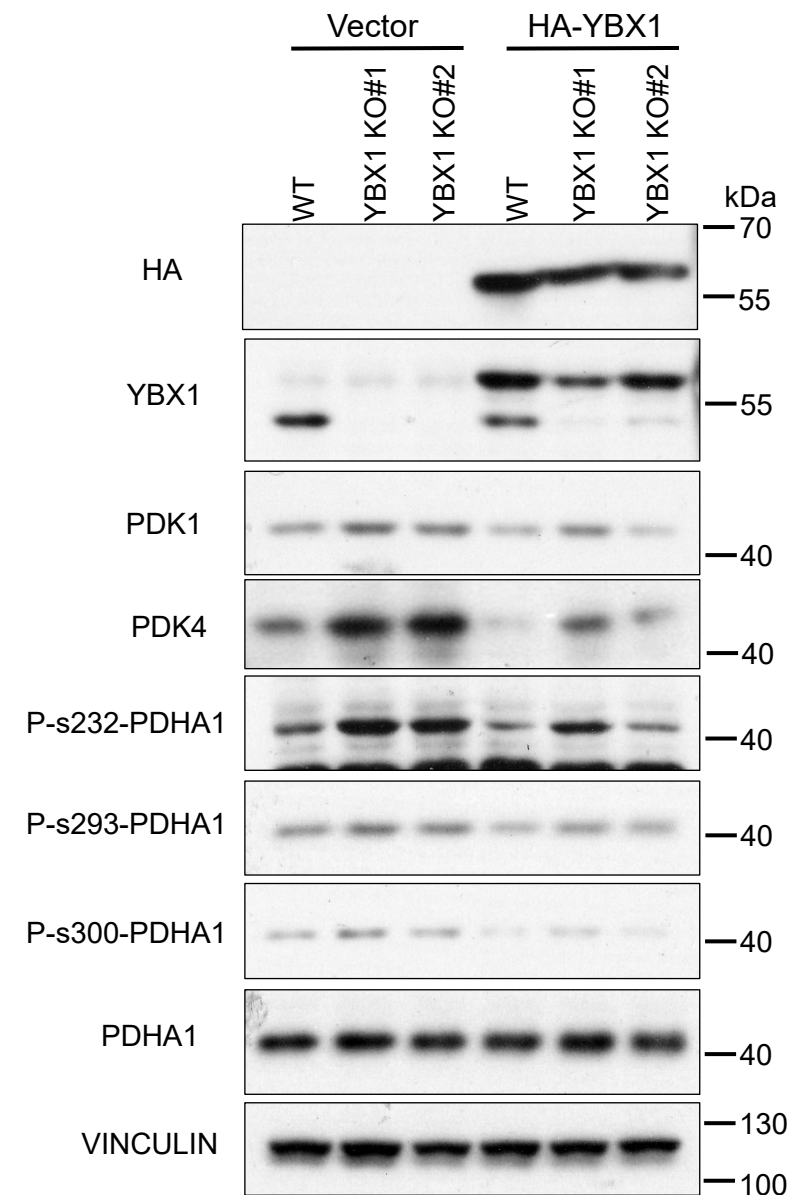

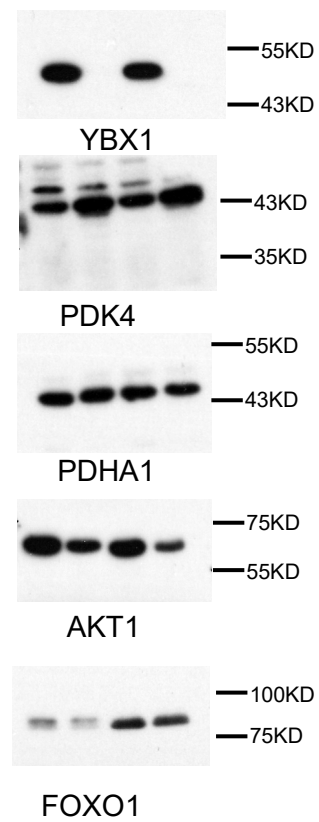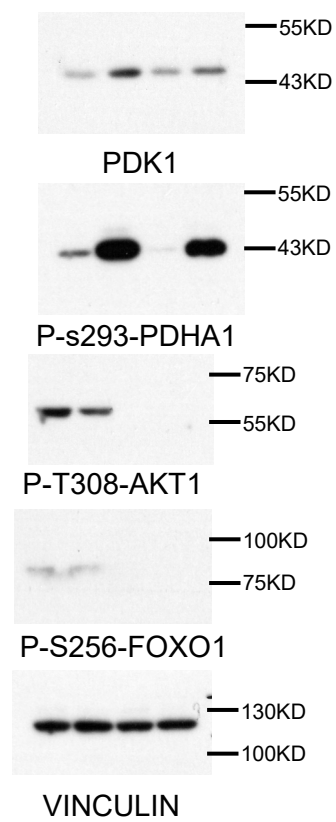

**Fig.s3 B**

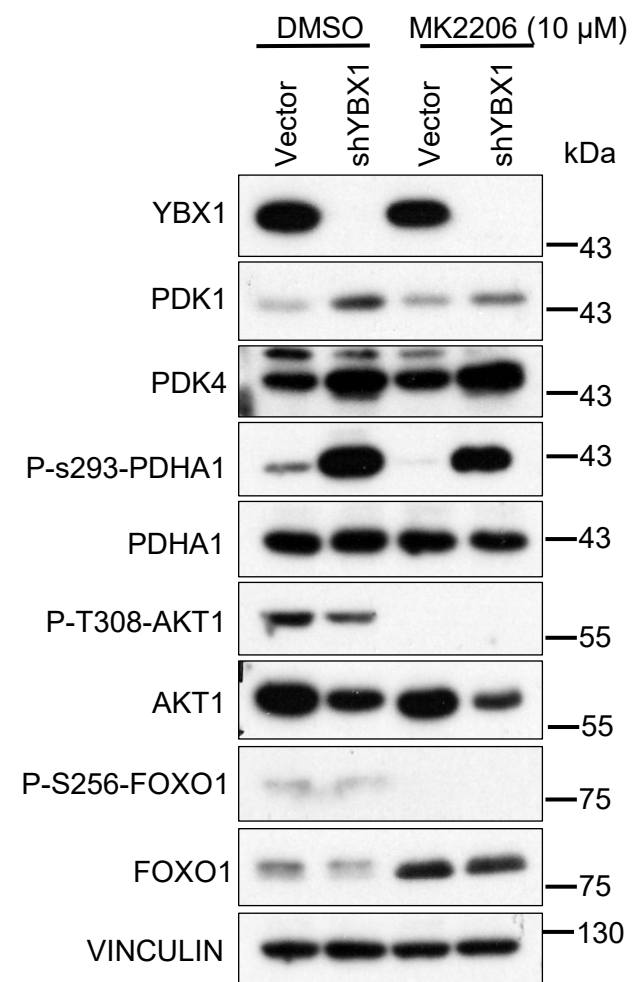

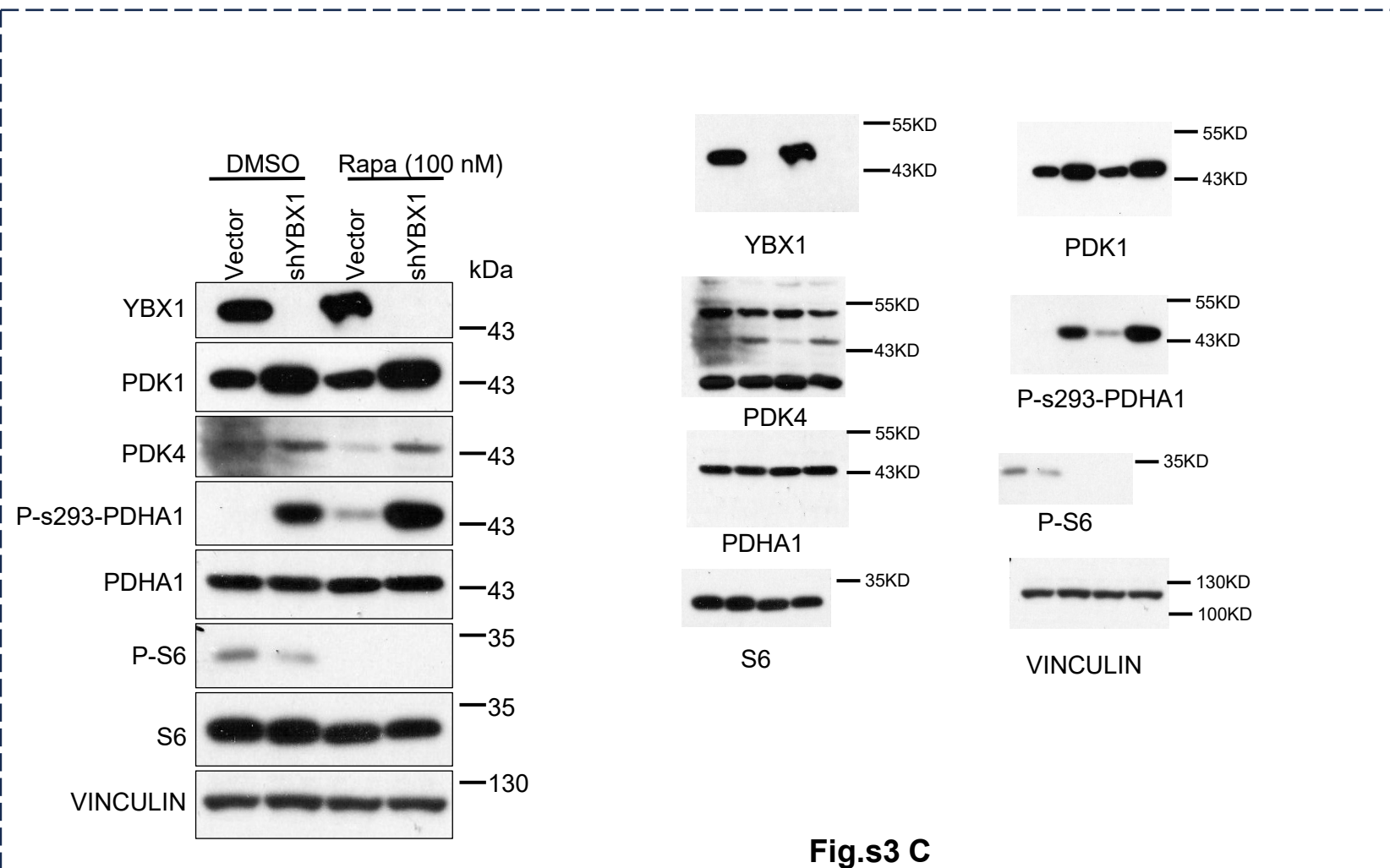

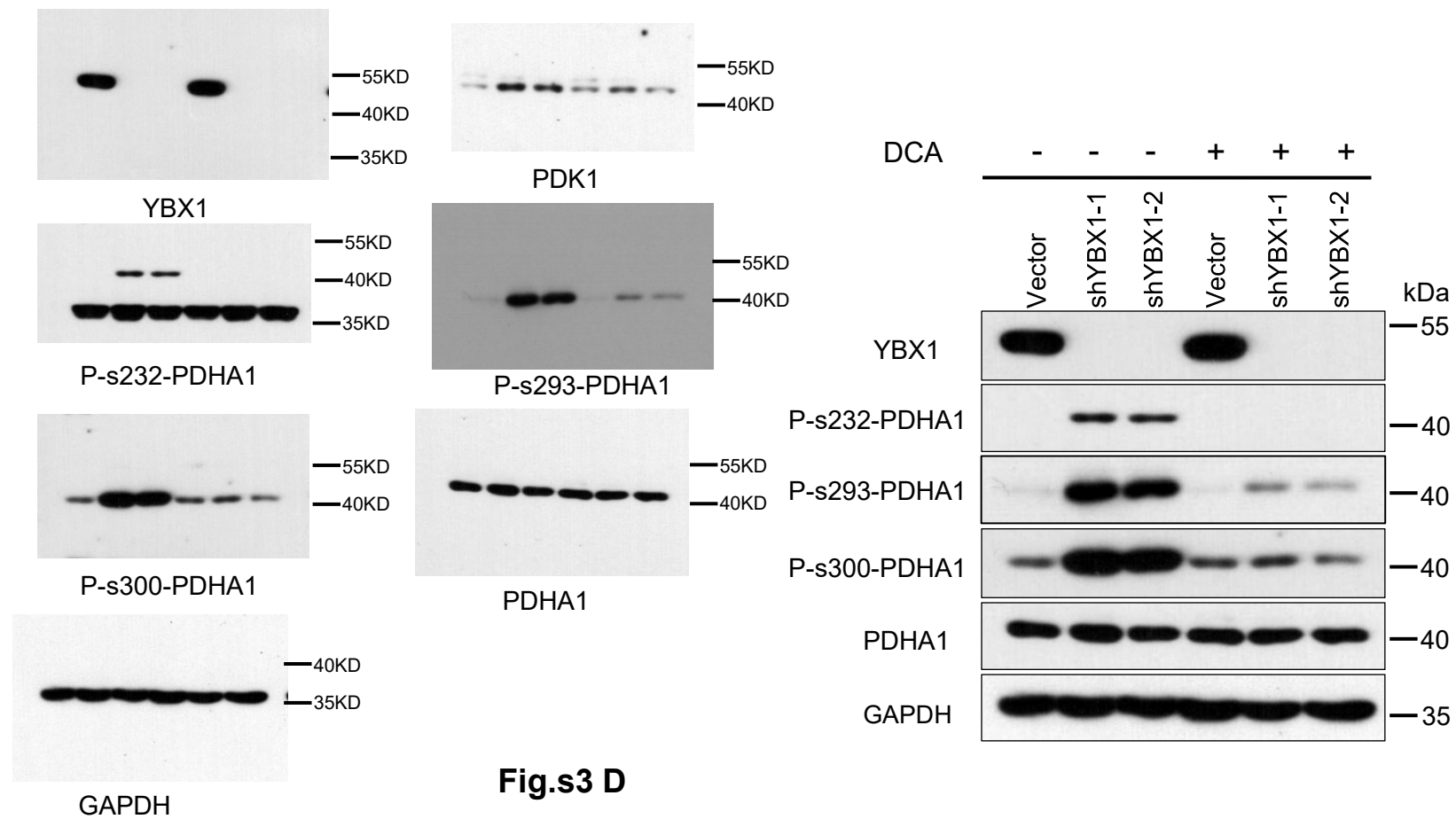

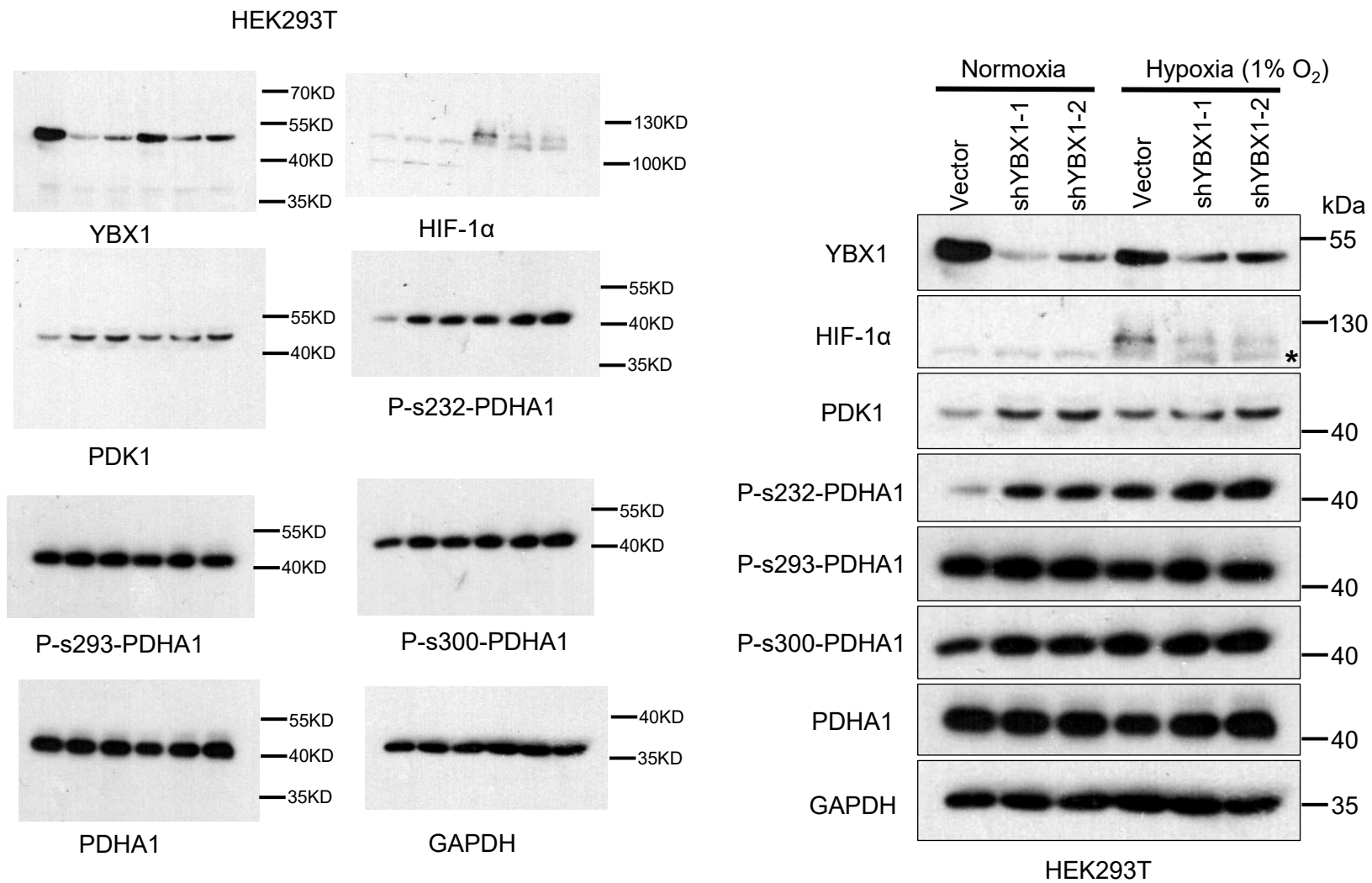

**Fig.4 A**

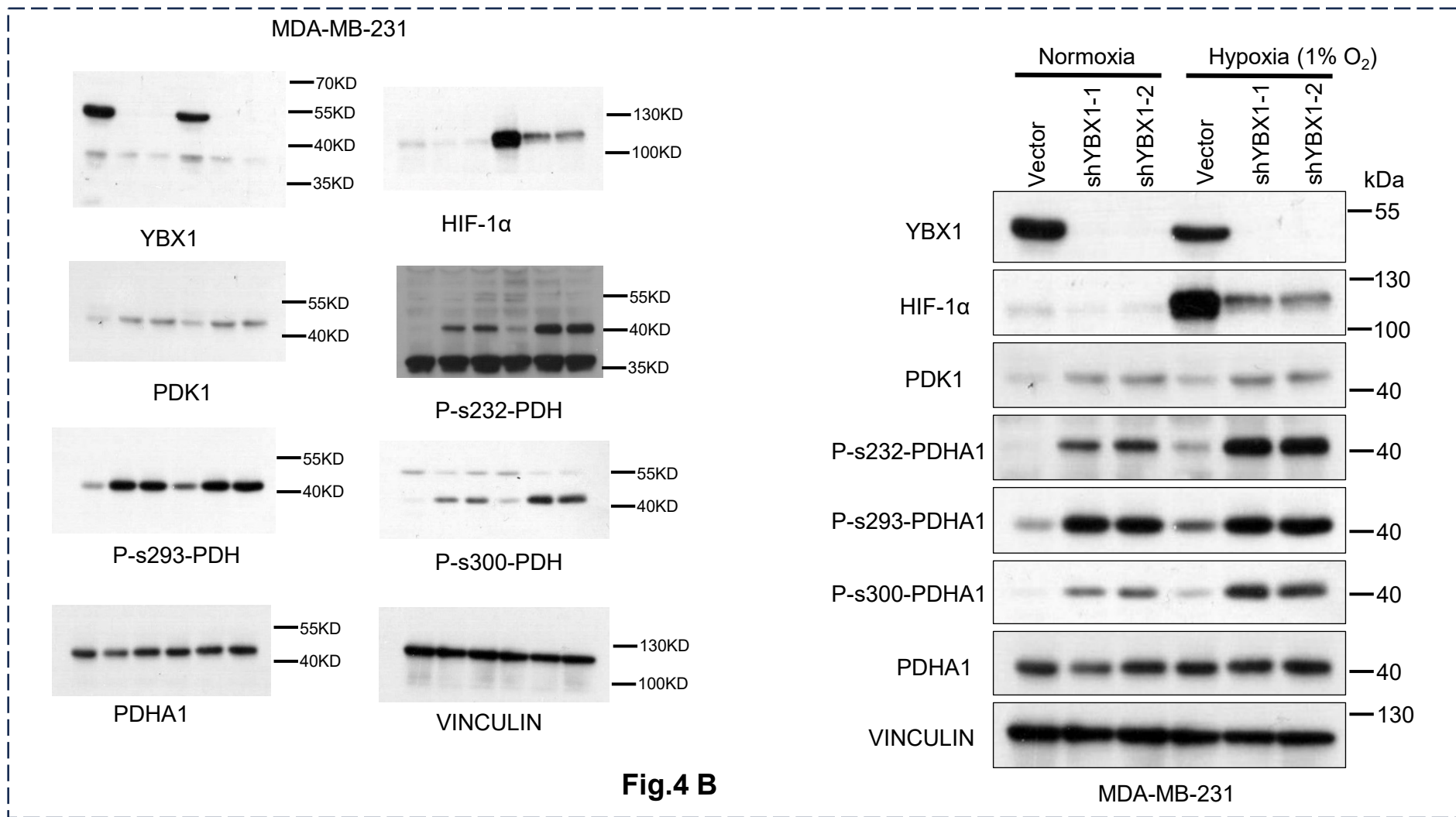

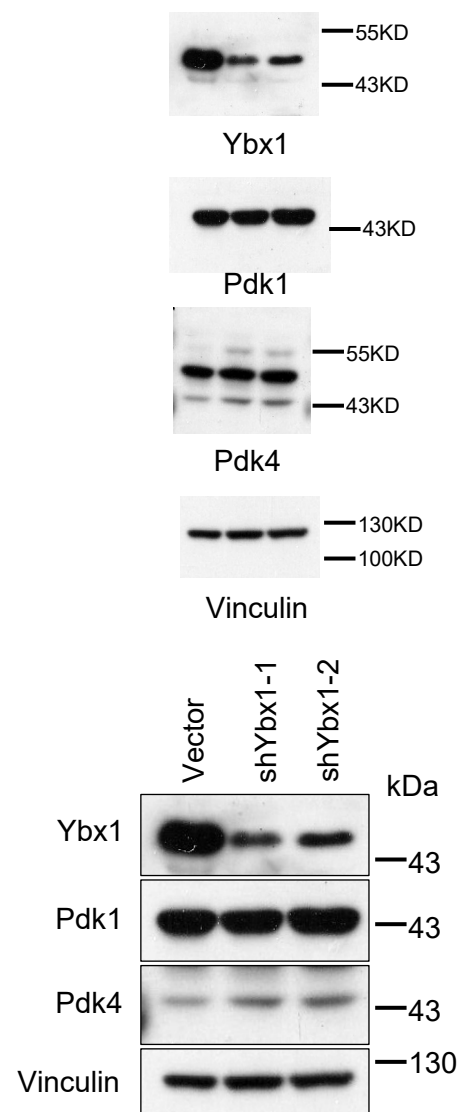

**Fig. s4 C**

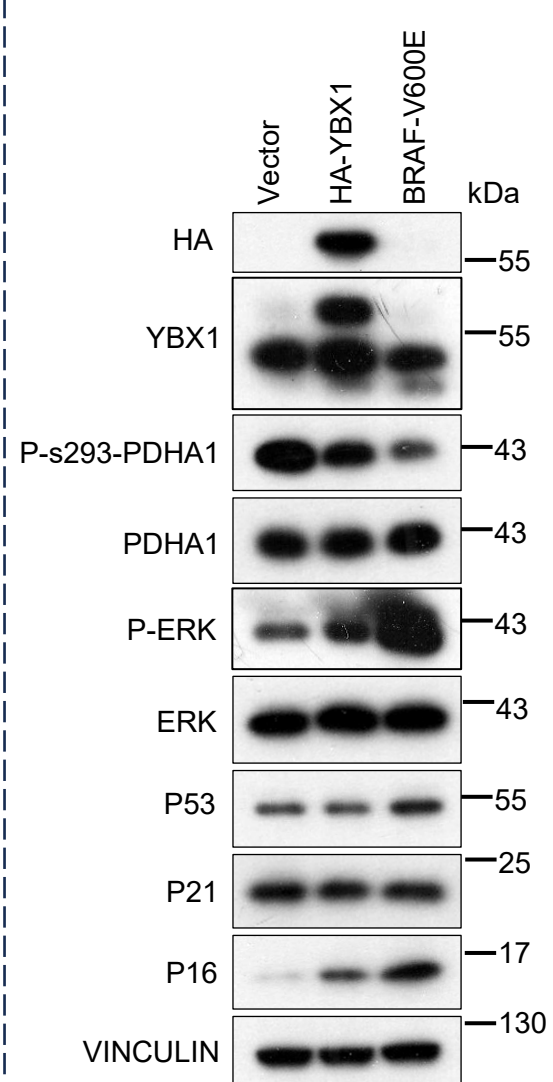

**Fig. 5 A**

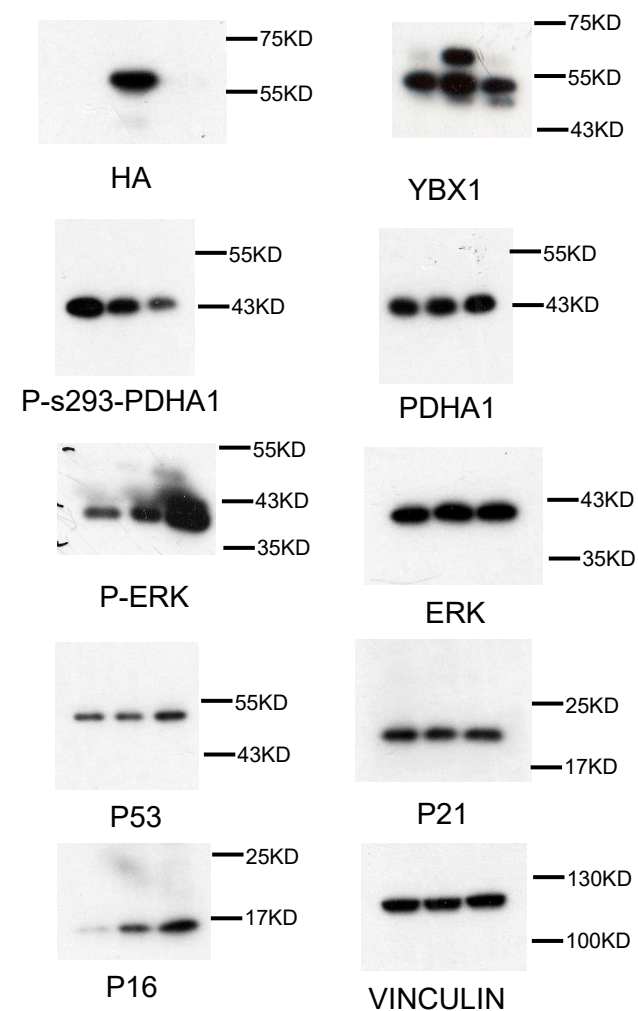

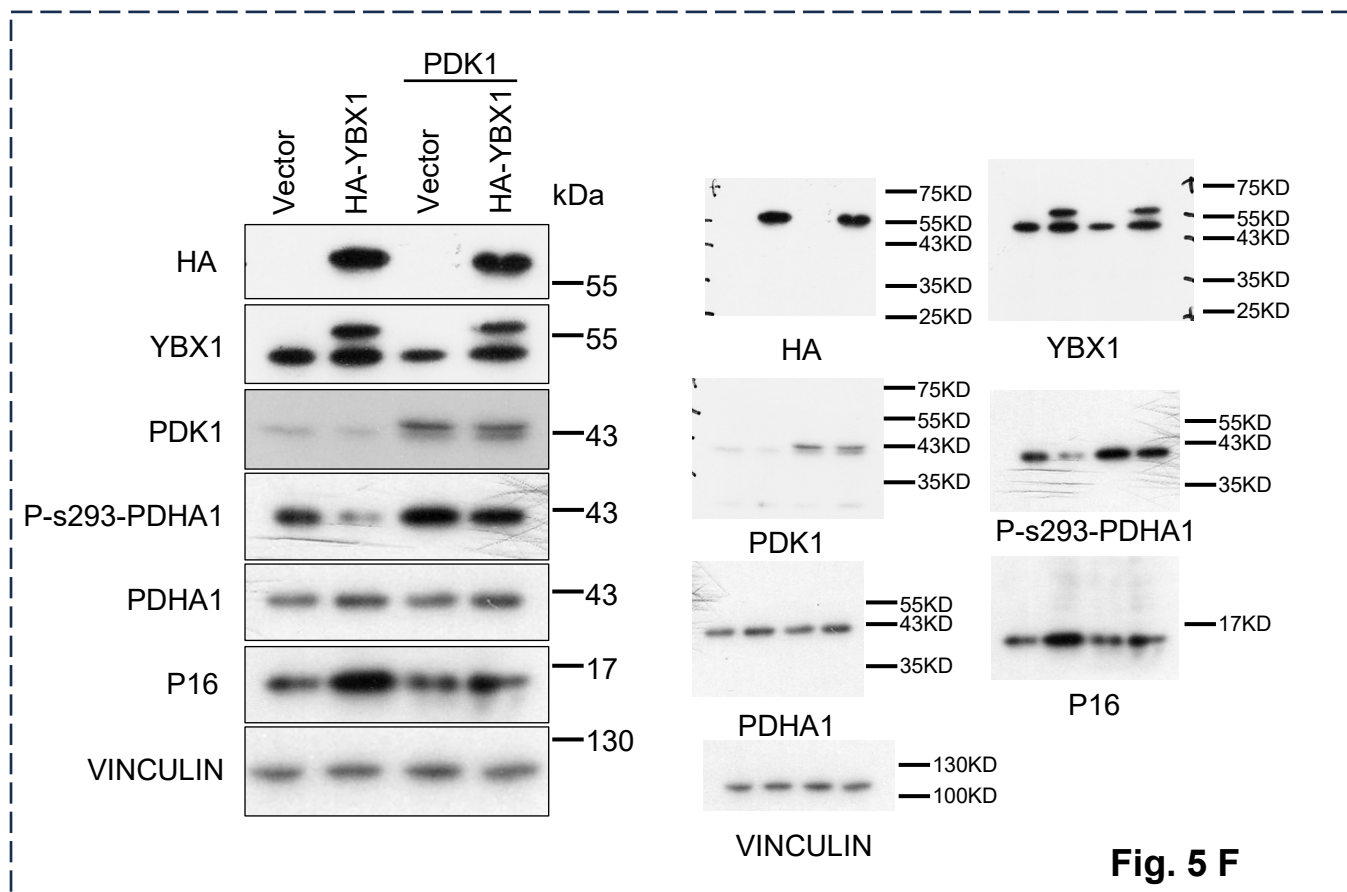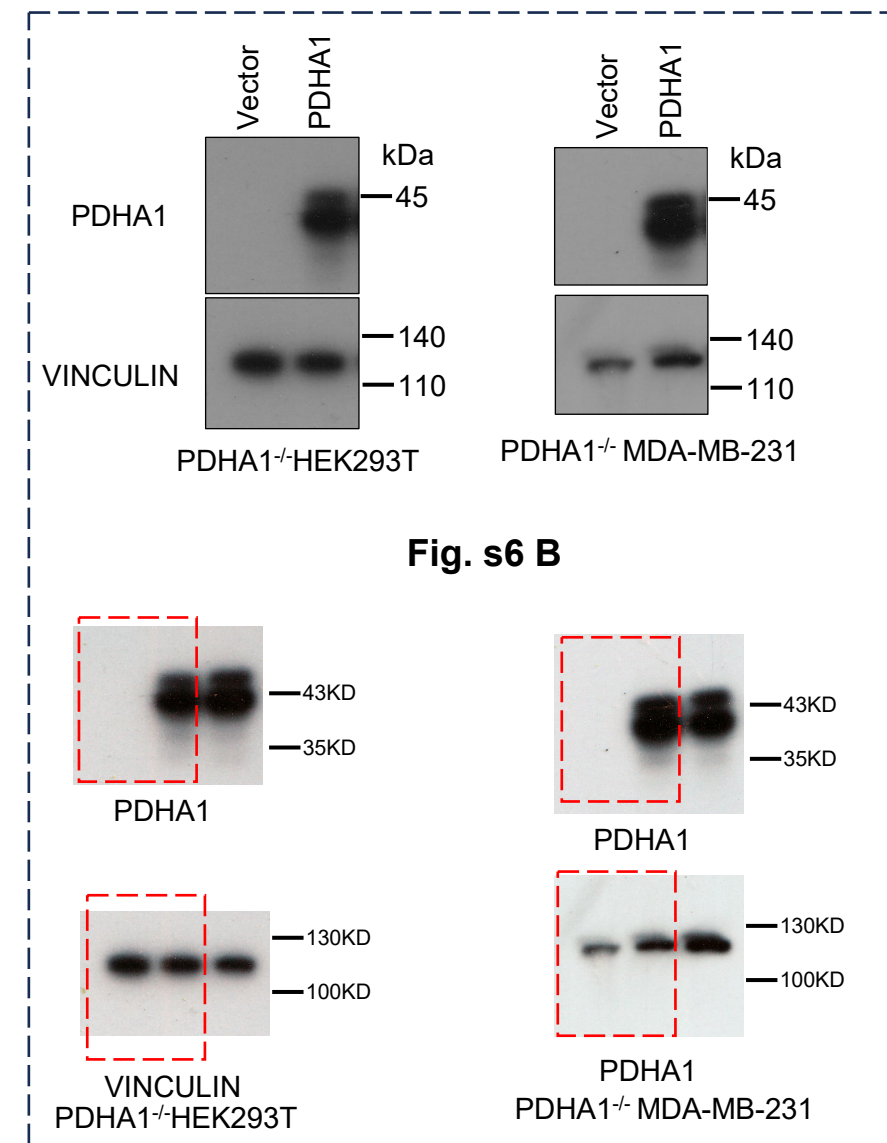

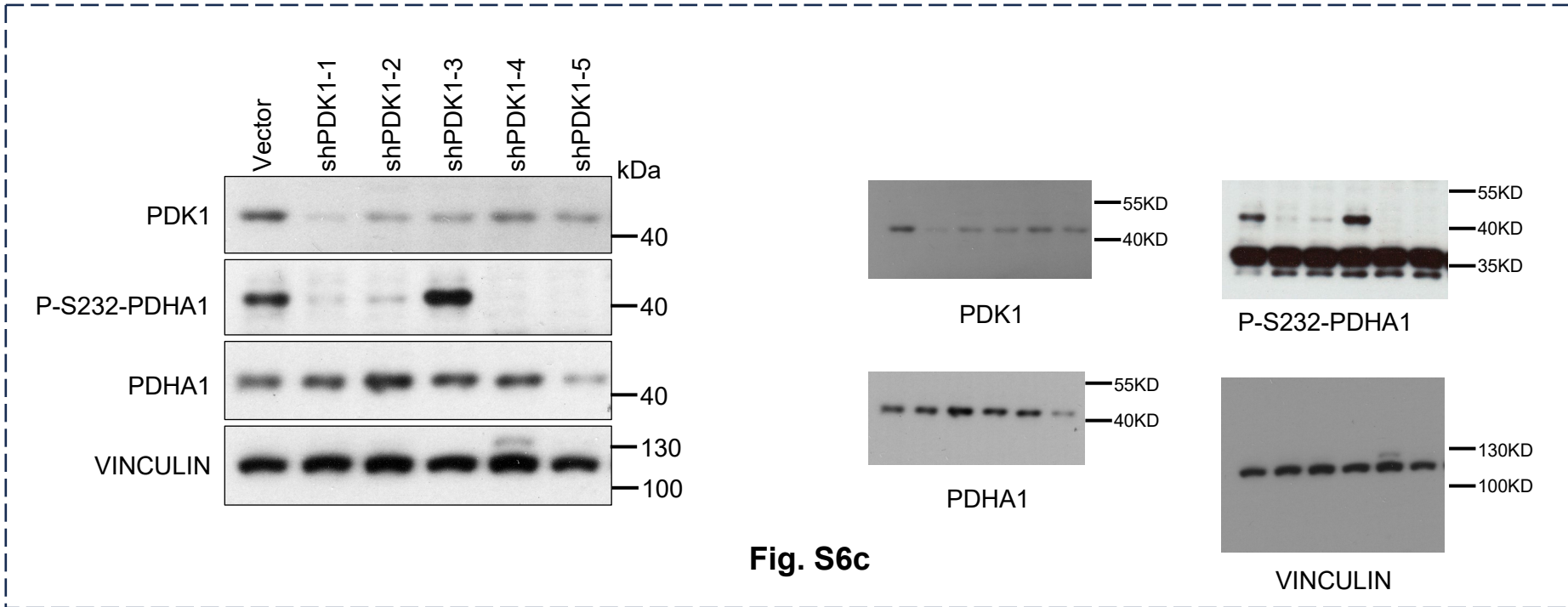



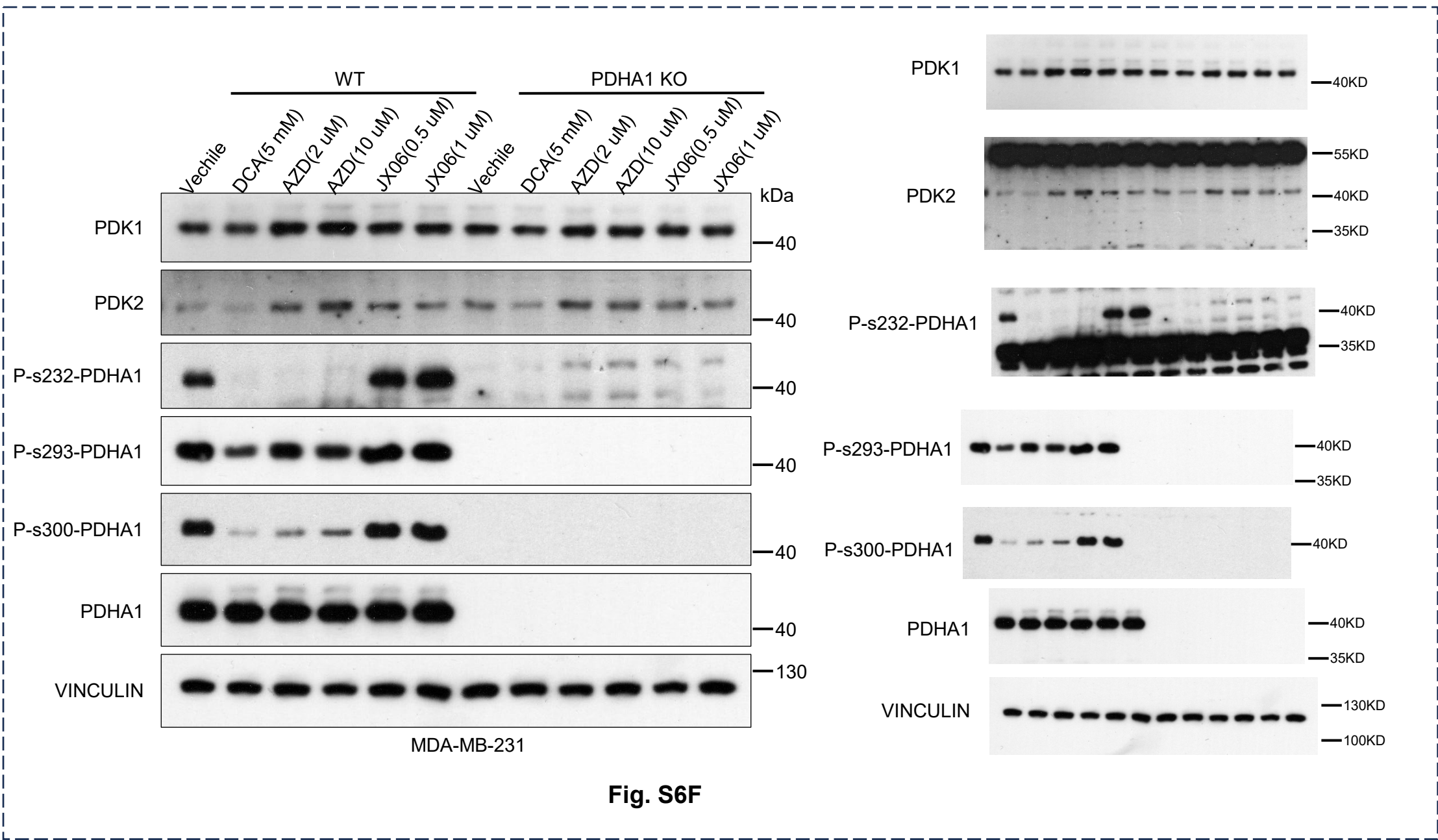

Supplement: Supplementary Data 1 [file mmc3.pdf]
