# Supplementary material for: The pyruvate dehydrogenase complex in concert with the DNA/RNA-binding protein YBX1 regulates cell senescence and tumorigenesis
Source: J Biol Chem. 2025 Aug 12;301(9):110585. doi: 10.1016/j.jbc.2025.110585 (PMC12446528; doi:10.1016/j.jbc.2025.110585)
Supplement: Supplementary Data 2 [file mmc4.docx]

**Supplementary Figures**

**
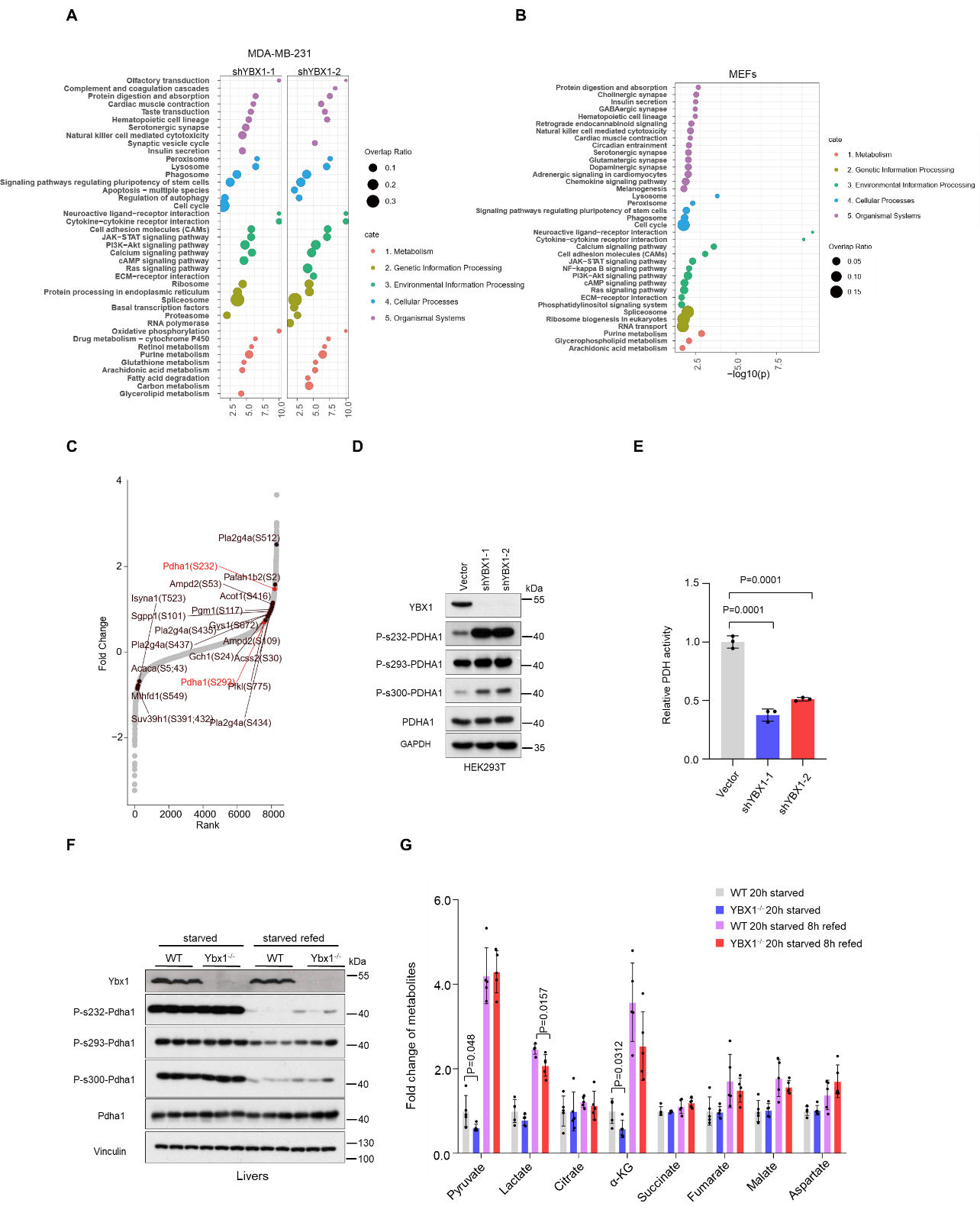
Supplementary Figure. 1** **PDHA1 activation required YBX1.**

(A and B) Enriched metabolic signaling pathway analysis based on significantly altered phosphoproteins in YBX1 knockdown MDA-MB-231 (a) and MEFs cells (b).

(C) Top ranked phosphosites related to metabolism in MEF cells were highlighted. Posphosites of Pdha1 were marked in red.

(D and E) HEK293T cells transduced with two independent YBX1 shRNAs and cell lysates were subjected to immunoblotting with indicated antibodies (d) and PDH enzymatic activity (*n*=3 biological independent samples) (e).

(F) Immunoblotting analysis of indicated antibodies in WT or *Ybx1*^-/-^ mice liver tissues. Starved: fasted for 20 h; Starved refed: fasted 12 hours and refed for another 8 hours.

(G) Sum of all fractions from related metabolites in Fig. 1i.

Results are the mean of biological replicates from a representative experiment, and error bars indicate s.d. Statistical significance was determined by a two-tailed, unpaired Student’s t-test. All experiments were repeated independently at least three times.

**
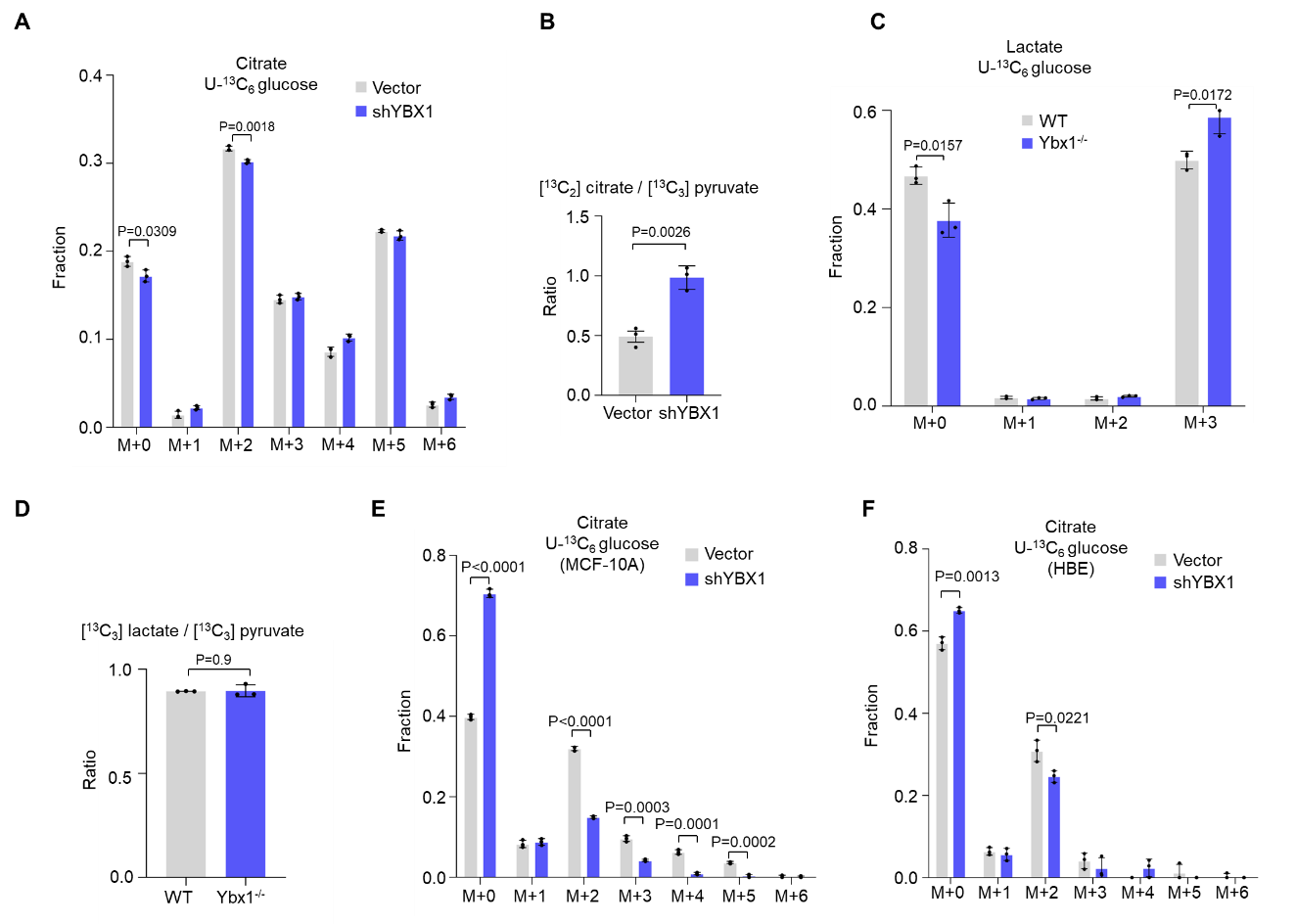
**

**Supplementary Figure. 2 YBX1 deficiency inactivates PDHA1 and impairs pyruvate oxidation.**

(A) Mass isotopologue analysis of citrate in vector and shRNA-mediated YBX1 knockdown cells incubated with [U-^13^C] glucose (*n*=3 biological independent samples).

(B) Mass isotopologue analysis of [^13^C_3_] citrate / [^13^C_3_] pyruvate in vector and shRNA-mediated YBX1 knockdown cells incubated with [U-^13^C] glucose (*n*=3 biological independent samples).

(C) Mass isotopologue analysis of lactate in wild-type (WT) and *Ybx*1^-/-^ MEFs incubated with [U-^13^C] glucose (*n*=3 biological independent samples).

(D) Mass isotopologue analysis of [^13^C_3_] lactate / [^13^C_3_] pyruvate in wild-type (WT) and *Ybx*1^-/-^ MEFs incubated with [U-^13^C] glucose (*n*=3 biological independent samples).

(E and F) Mass isotopologue analysis of citrate in HBE (e) and MCF-10A (f) cells transduced with YBX1 shRNA incubated with [U-^13^C] glucose (*n*=3 biological independent samples).

Results in (A-F) are the mean of biological replicates from a representative experiment, and error bars indicate s.d. Statistical significance was determined by a two-tailed, unpaired Student’s t-test. All experiments were repeated independently at least three times.

**
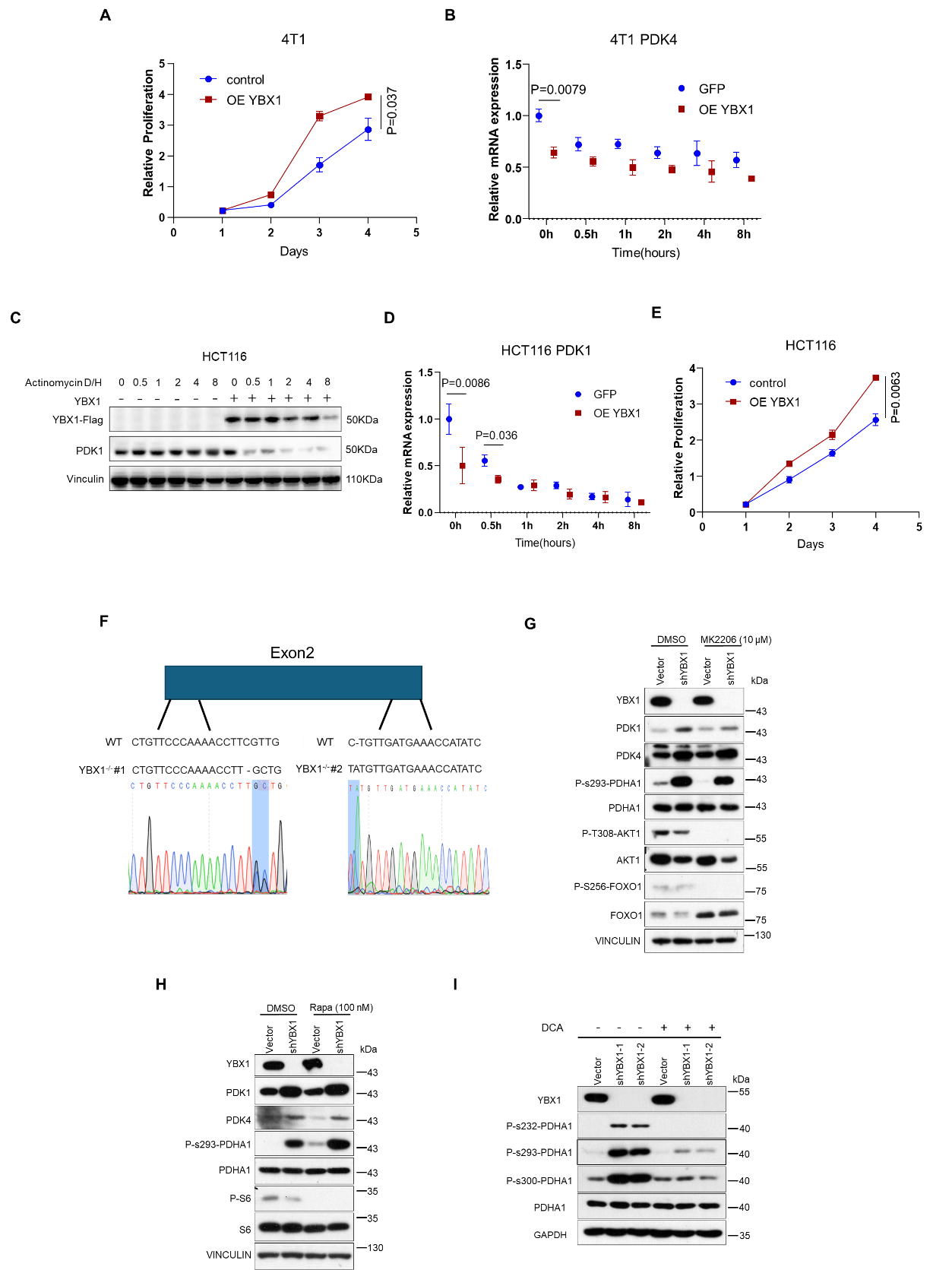
**

**Supplementary Figure. 3 YBX1 regulates** **pyruvate dehydrogenase kinase.**

(A) Relative cell growth curve of control and YBX1 over expressed 4T1 cells (n=5 biological independent samples).

(B) qRT-PCR analysis of PDK4 mRNA expression levels in 4T1 cells under Actinomycin D (5 μM) treatment at different time points (0-8 hours) (n=4 biological independent samples).

(C) Immunoblot analysis of HCT116 cells transduced with either control or YBX1 overexpressing plasmids. Cells were treated with Actinomycin D (5 μM) for the indicated time points (0–8hours). Representative images from three independent experiments are shown.

(D) qRT-PCR analysis of PDK1 mRNA expression levels in HCT116 cells under Actinomycin D (5 μM) treatment at different time points (0-8 hours) (n=4 biological independent samples).

(E) Relative cell growth curve of control and YBX1 over expressed HCT116 cells (n=5 biological independent samples).

(F) Diagram of two clones of CRISPR/Cas9-mediated YBX1 knockout (KO) in MDA-MB-231 cells.

(G) Immunoblotting analysis of indicated proteins in MDA-MB-231 cells transduced with control shRNA or YBX1 shRNAs. Treatment with MK2206(10 μM) for 24 h.

(H) Immunoblotting analysis of indicated proteins in MDA-MB-231 cells transduced with control shRNA or YBX1 shRNAs. Treatment with Rapamycin (100 nM) for 12 h.

(I) Immunoblotting analysis of indicated proteins in MDA-MB-231 cells transduced with control shRNA or two independent YBX1 shRNAs. Treatment with DCA (5 mM) for 6 h.

Results in A, B, D and E are the Mean ± SEM of biological replicates from a representative experiment, and error bars indicate s.d. Statistical significance was determined by a two-tailed, unpaired Student’s t-test.


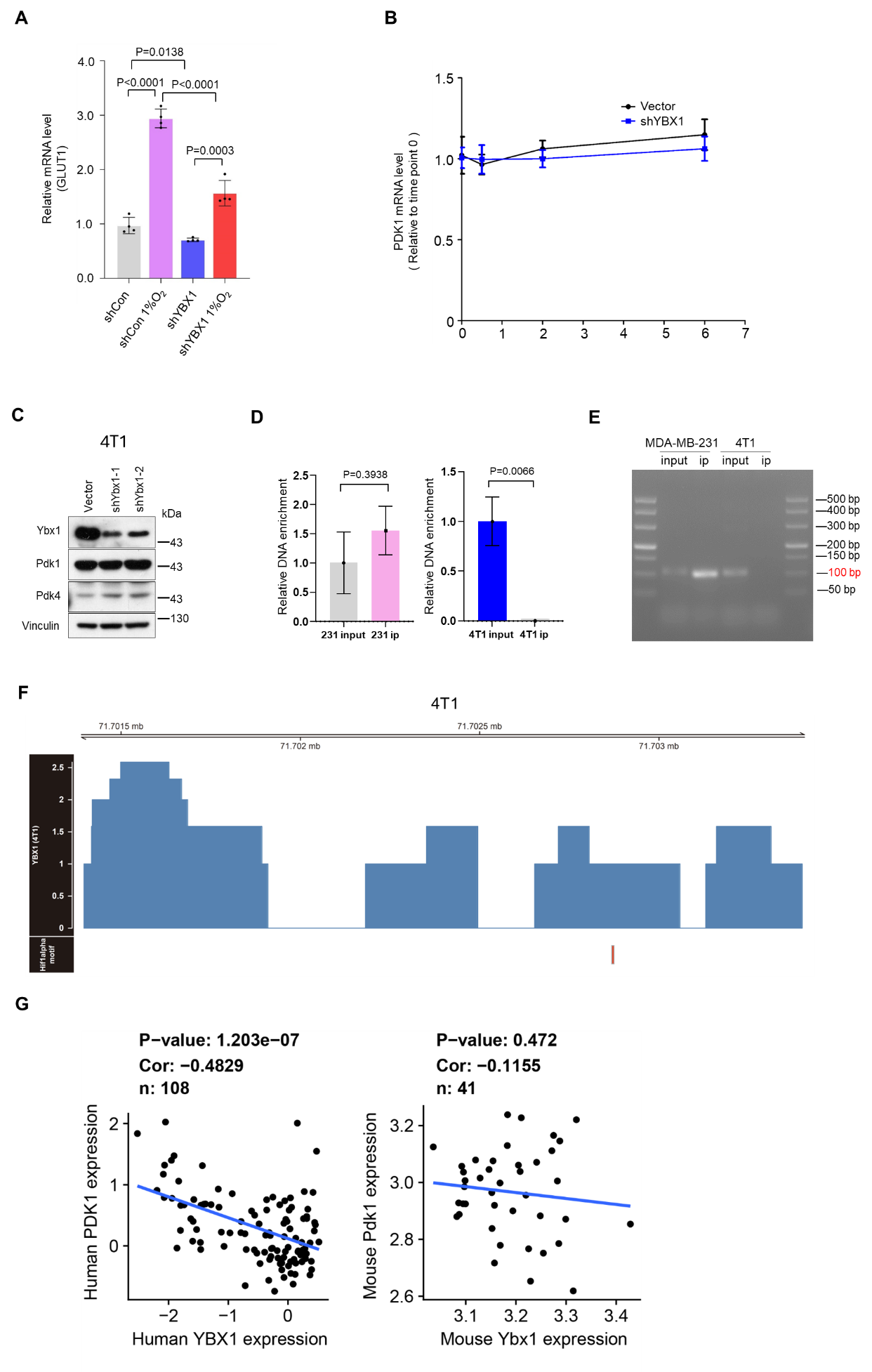


**Supplementary Figure. 4** **YBX1 inhibits PDK1 expression.**

(A) qRT-PCR analysis of *GLUT1* mRNA expression levels in HEK293T cells under normoxia or hypoxia treatment (6 hours) (*n*=4 biological independent samples).

(B) Transcript degradation rate analysis of *PDK1* in MDA-MB-231 cells under actinomycin D treatment (*n*=3 biological independent samples).

(C) Immunoblotting analysis of indicated proteins in mouse 4T1 cells transduced with control shRNA or two independent YBX1 shRNAs.

(D) ChIP-qPCR analysis of YBX1 enrichment at the PDK1 promoter in MDA-MB-231 and 4T1 cells. Chromatin was sonicated and immunoprecipitated with an anti-YBX1 antibody. The precipitated DNA was analyzed by qPCR using primers flanking the CAGGACTTCCTG motif. Ip Data are presented as fold enrichment over input (mean ± SEM, Student’s t-test, n=4 biological independent samples).

(E) PCR validation of the immunoprecipitated DNA in MDA-MB-231 and 4T1 cells. Agarose gel shows amplification of the PDK1 promoter region containing the CAGGACTTCCTG motif in YBX1 ChIP samples (Input and anti-YBX1).

(F) Genomic browser tracks showing YBX1 ChIP-seq signal peaks (p < 0.001, MACS2) at the PDK1 promoter locus (chr2:71,701,398-71,703,398) in 4T1 cells. Highlighted regions indicate conserved binding motifs: HIF1α (CACGTG, red).

(G) Scatter plots correlation analysis of *YBX1* and *PDK1* based on the TCGA database. Cor: represents the pearson correlation coefficient.

Results in (A) and (B) are the mean of biological replicates from a representative experiment, and error bars indicate s.d. Statistical significance was determined by a two-tailed, unpaired Student’s t-test. All experiments were repeated independently at least three times.


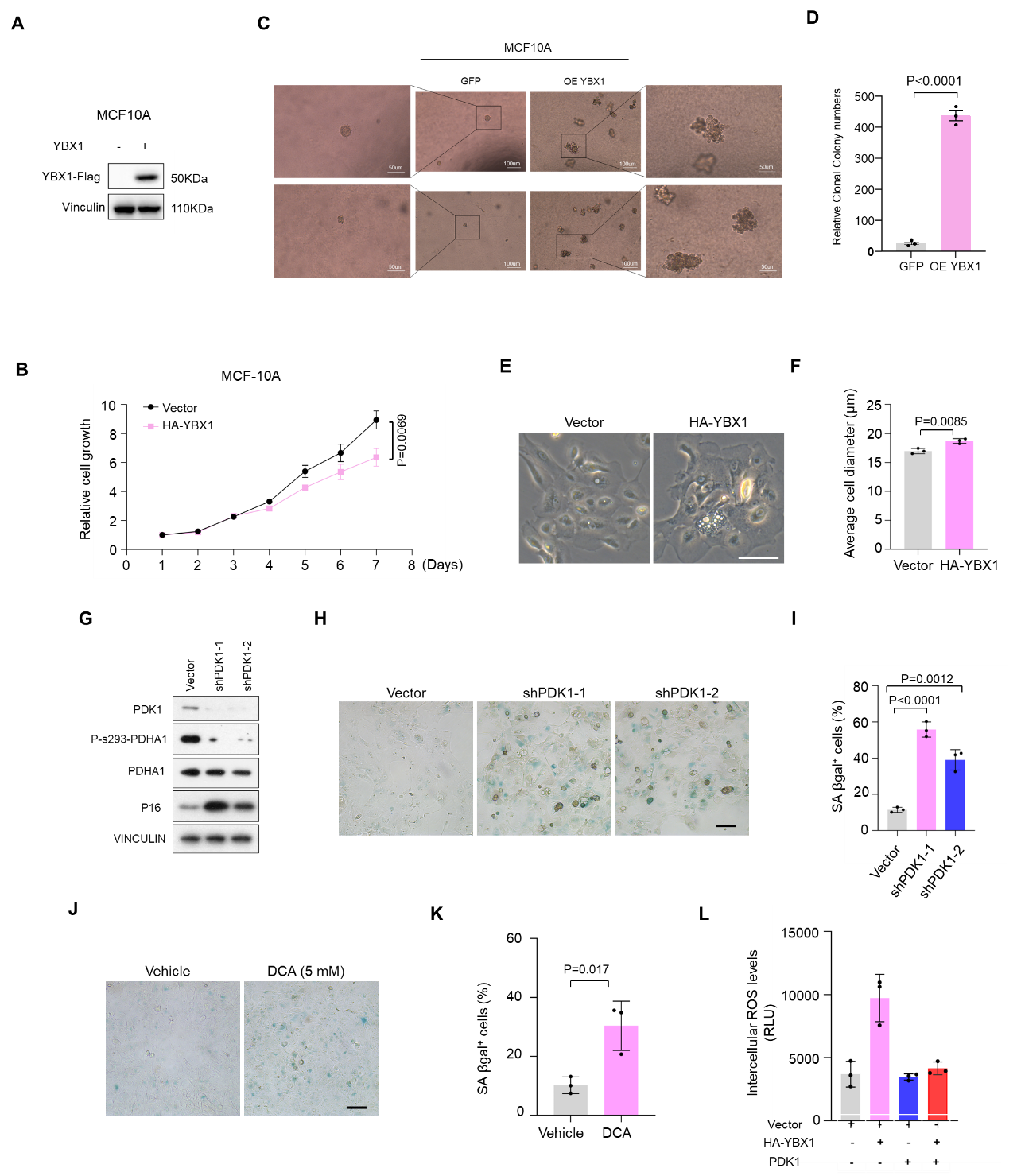


**Supplementary Figure. 5 YBX1 induces senescence by regulating PDHA1 activity**

(A) Immunoblot analysis of MCF10A cells transduced with either control GFP or YBX1-Flag overexpressing constructs. Representative images from three independent experiments are shown.

(B) Relative growth curve of vector and HA-tagged YBX1 MCF-10A cells (*n*=3 biological independent samples).

(C) Representative micrographs of soft agar colony formation assay (scale bar: 50 μm or 100um).

(D) Statistical analysis of colony area distribution in MCF10A cells. Cells were seeded in 0.3% ~ 0.75% soft agar and cultured for 14 days. Colony areas were quantified using ImageJ software. Data represent mean ± SEM from three independent experiments (P<0.0001 by two tailed, unpaired t-test, OE, overexpression group).

(E) Representative images of vector and HA-tagged YBX1 MCF-10A cells.

(F) Quantification of vector and HA-tagged YBX1 MCF-10A cell diameter using Countess 3 (*n*=3 biological independent samples).

(G) Immunoblotting analysis of indicated proteins in MCF10A cells transduced with control shRNA or PDK1 shRNAs.

(H and I) Representative images and quantification of SA-β-gal–positive cells in MCF-10A cells transduced with two independent PDK1 shRNAs . Scal bars, 100 μm.

(J and K) Representative images and quantification of SA-β-gal–positive cells in MCF10A cells under DCA treatment. Scal bars, 100 μm; DCA treatment for 9 days.

(L) Detection of intercellular ROS of vector, HA-tagged YBX1, PDK1 and HA-tagged YBX1 combined with PDK1 in MCF-10A cells (*n*=3 biological independent samples).

Results are the mean of biological replicates from a representative experiment, and error bars indicate s.d. Statistical significance was determined by a two-tailed, unpaired Student’s t-test. All experiments were repeated independently at least three times.


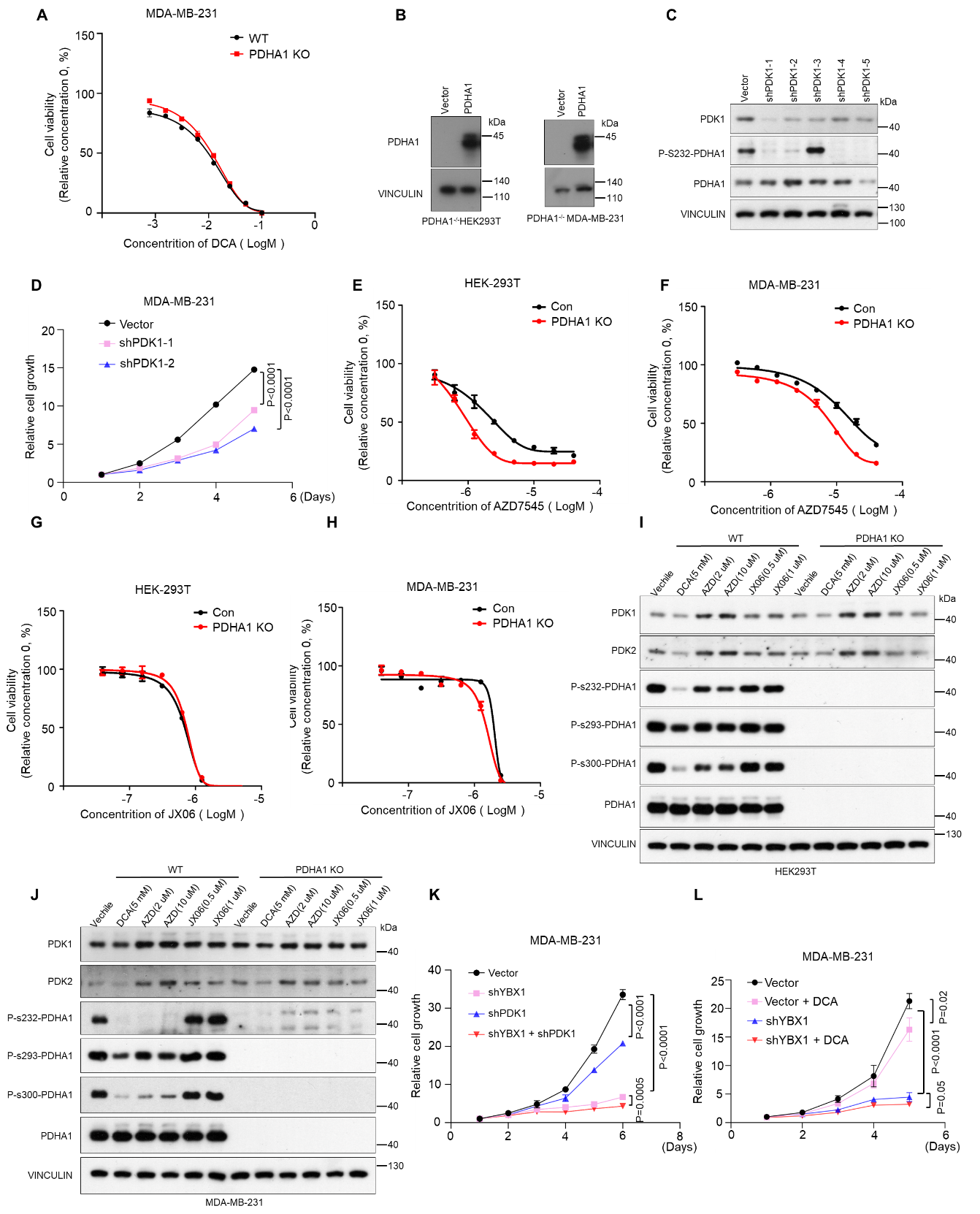


**Supplementary Figure. 6 PDHA1 activation constrain tumorigenic capacity of YBX1.**

(A) The cell viability of WT and PDHA1 knockout MDA-MB-231 cells treated with different concentrations (0, 0.78125, 1.5625, 3.125, 6.25, 12.5, 25, 50, 100; mM) of DCA for 96 hours. (*n*=3 biological independent samples).

(B) Immunoblotting analysis of PDHA1 and VINLULIN in PDHA1^-/-^ and PDHA1 restoration HEK293T and MDA-MB-231 cells.

(C) Immunoblotting analysis of PDK1 shRNA efficiency in MDA-MB-231 cells.

(D) Relative growth curve of vector and shRNA-mediated PDK1 knockdown MDA-MB-231 cells (*n*=3 biological independent samples).

(E and F) The cell viability of Control and PDHA1 knockout HEK293T (E) and MDA-MB-231 (F) cells treated with different concentrations (0, 0.3125, 0.625, 1.25, 2.5, 5, 10, 20, 40; μM) of AZD7545 for 96 hours. (*n*=3 biological independent samples).

(G and H) The cell viability of Control and PDHA1 knockout HEK293T (G) and MDA-MB-231 (H) cells treated with different concentrations (0, 0.039063, 0.078125, 0.15625, 0.3125, 0.625, 1.25, 2.5, 5; μM) of JX06 for 72 hours. (*n*=3 biological independent samples).

(I and J) Immunoblotting analysis of indicated proteins in HEK293T (I) and MDA-MB-231(J) cells incubated with DCA (5 mM), AZD7545 (2, 10; mM) or JX06 (0.5, 1; μM) for 12 hours.

(K) Relative growth curve of vector, shYBX1, shPDK1 and shYBX1 combined with shPDK1 MDA-MB-231 cells (*n*=3 biological independent samples).

(L) Relative growth curve of vector and shYBX1 combined with or without DCA (5 mM) treatment in MDA-MB-231 cells (*n*=3 biological independent samples).

Results in (a) and (d-f) are the mean of biological replicates from a representative experiment, and error bars indicate s.d. Statistical significance was determined by a two-tailed, unpaired Student’s t-test. All experiments were repeated independently at least three times.
